# Pheromone-Binding Proteins in Pest Control: From Molecular Insights to Real-World Applications

**DOI:** 10.1021/acs.jafc.5c03663

**Published:** 2025-08-21

**Authors:** Ishani Ray, Smita Mohanty

**Affiliations:** Department of Chemistry, 33086 Oklahoma State University, Stillwater, Oklahoma 74078, United States

**Keywords:** pheromone-binding proteins (PBPs), olfaction in insects, lepidopteran sex pheromones, ligand binding, olfactory receptors (ORs), odorant-binding proteins (OBPs), signal transduction, integrated pest management (IPM), pest control strategies

## Abstract

Insects utilize sophisticated olfactory systems to detect chemical cues critical for behaviors such as mating, host selection, and predator avoidance. In lepidopteran moths, sex pheromone communication offers a well-established model in which males detect female-emitted signals over long distances. Central to this process are pheromone-binding proteins (PBPs), which solubilize and transport hydrophobic pheromones through the sensillar lymph to olfactory receptors, enabling precise signal detection. Recent advances in molecular biology, structural biochemistry, and gene-editing technologies such as CRISPR/Cas9 have uncovered nuanced mechanisms underlying PBP function, including ligand-binding specificity, pH-dependent conformational dynamics, and molecular interactions. These discoveries have broad implications, extending beyond chemosensory biology to applications in reverse chemical ecology, biosensing, and environmentally conscious pest control. This review synthesizes insights from *in vitro*, *in silico*, and *in vivo* studies, highlighting the structural and functional diversity of PBPs across species and emphasizing their translational utility as molecular targets for sustainable agriculture and biodiversity conservation.

## Graphical abstract

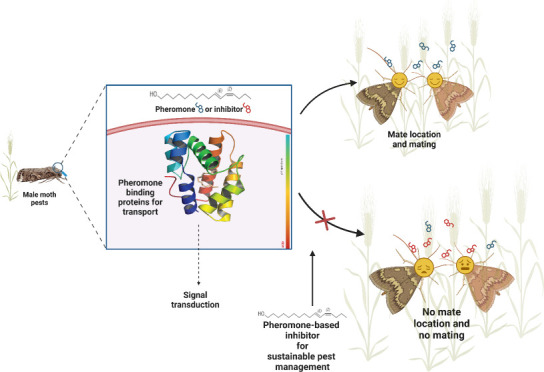

## Introduction

Insects have evolved extremely sensitive olfactory systems to decode an array of environmental chemical cues that regulate behaviors such as mating, host location, and predator avoidance.^1,2^ Among these, sex pheromone-mediated communication, where males detect sex pheromones released by conspecific females over long distances, is particularly well-characterized in lepidopteran species, offering a model system for understanding olfactory signal transduction.^3,4^ Central to this process are pheromone-binding proteins (PBPs), a specialized subclass of odorant-binding proteins (OBPs) that facilitate the solubilization and transport of hydrophobic pheromones through the aqueous sensillar lymph to specific olfactory receptors (ORs).^5,6^ Acting as molecular filters, PBPs ensure specificity in pheromone detection, playing an essential role in species recognition and reproductive success.^6^

While PBPs have been most extensively studied in Lepidoptera, they are also present across insect orders—including beetles (Coleoptera), cockroaches (Blattodea), and flies (Diptera)—where they support pheromone or odorant detection in species-specific contexts.^7,8^ For example, PBPs in *Periplaneta americana* bind sex and aggregation pheromones with nanomolar affinity for mating and social behaviors.^9^ Similarly, in *Drosophila melanogaster*, OBP76a (LUSH) mediates detection of the courtship pheromone cis-vaccenyl acetate (cVA).^10^ These examples underscore a conserved chemosensory role for PBPs and related OBPs, although their structural motifs and ecological functions exhibit considerable variation across insect lineages.^7,8^

This perspective presents a comprehensive synthesis of current knowledge on lepidopteran PBPs, where structure–function relationships are most thoroughly characterized across insect orders. We explore the structural and functional diversity of these PBPs, their ligand-binding mechanisms, and their emerging applications in pest management. By integrating evidence from *in vitro*, *in silico*, and *in vivo* studies, we highlight the central role of PBPs as molecular gatekeepers of insect olfaction and as promising targets for the development of next-generation strategies in sustainable agricultural and ecological management.

### 1.1 Olfaction and the Insect Olfactory System

Olfaction is fundamental to the survival of both vertebrates and invertebrates, with chemical compounds serving as vital information carriers.^8^ Chemoreception guides essential behaviors such as foraging, host location, alarm signaling, courtship, taxis, locating oviposition sites, and predator avoidance.^2,8^ Invertebrates, particularly insects, rely heavily on olfaction as their primary sensory modality, using it to detect and discriminate trace chemical cues with exceptional sensitivity, speed, and specificity.^1,2,8^ Understanding the signal transduction mechanisms underlying invertebrate olfaction not only provides insights into fundamental principles of chemosensation and the progression from olfactory information to behavioral response but also informs the understanding of sensory processing in higher organisms across taxa.

The insect olfactory system provides a model framework for studying olfaction. While it shares some structural and functional parallels with vertebrate systems, it features unique anatomical and physiological adaptations enabling the detection of semiochemicals, small, hydrophobic molecules that facilitate intra- and interspecies communication.^11^ A well-characterized example is the sex pheromone detection system in lepidopteran moths. The order Lepidoptera, comprising moths and butterflies, is the second largest insect order following Coleoptera and includes numerous species with highly specialized pheromone-mediated signaling pathways.^12,13^ Many lepidopteran species are of considerable economic importance, as several are among the most destructive agricultural pests globally, inflicting significant damage on staple and high-value crops.^14^ Male moths rely on sex pheromones released by conspecific females for precise mate location over long distances.^3,4^ Beyond pheromones, other classes of semiochemicals (allelochemicals), such as kairomones, synomones, and allomones, mediate interspecies interactions in moths, functioning as attractants or repellents depending on ecological context.^15^

In insects, olfactory perception is initiated at the antennae, which are covered with thousands of hair-like specialized sensory structures known as sensilla, finely tuned to detect airborne semiochemicals.^16^ Antennal morphology across moth species exhibits sexual dimorphism, with males possessing larger, plumose (feather-like, branched) structures to accommodate a greater density of sensory hairs.^17^ These sensilla enable insects to process a variety of semiochemicals, including host plant volatiles, pheromones, and sapid molecules via specific chemosensory receptors.^16^

A typical sensillum consists of a hollow, single-walled, cuticular hair (10–400 μm in length and 1–5 μm in diameter) penetrated with numerous pores (∼10–20 nm in diameter) allowing entry of odor molecules adsorbed on the surface^18^ (Figure 1). Inside, 1–4 olfactory sensory neurons (OSNs) or olfactory receptor neurons (ORNs) reside in a lymph-filled cavity and transduce chemical signals into electrical signals by modulating ion potentials across their plasma membranes.^16,19^ The sensillar lymph, secreted by auxiliary cells, protects the delicate OSN dendrites and is enriched in odorant-binding proteins (OBPs), pheromone-degrading enzymes (PDEs), and fatty acids like linoleic and palmitic acid.^16,20–26^ OSNs extend dendritic projections into the hair lumen, whereas their axons project to the macro glomerular complex in the antennal lobe of the central nervous system.^27^ Odorant detection occurs at the outer dendritic membrane, where olfactory receptors (ORs) are concentrated, with the dendritic region also featuring a ciliary segment (∼2 μm long) that separates the inner and outer dendritic segments for signal processing efficiency.^19^

**Figure 1:**
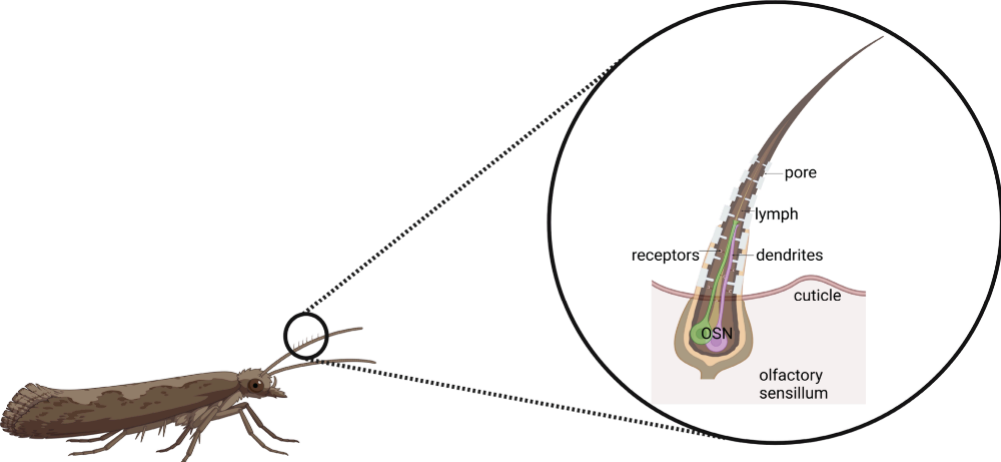
Moth olfactory sensillum structure. The image illustrates the moth’s antenna, which houses specialized olfactory sensilla responsible for detecting pheromones. A magnified cross-section of a sensillum shows its structural components, including cuticle pores, lymph, dendrites of olfactory sensory neurons, and olfactory receptors. Created in BioRender in collaboration with Smita Mohanty. Ray, I. (2025) https://BioRender.com/05ei6au.

Sensilla are categorized morphologically and functionally as basiconic, coeloconic, or trichoid.^27^ Among these, trichoid sensilla, specialized for pheromone detection, are abundant in males and absent or reduced in females.^28^ Basiconic sensilla detect general odors and other semiochemicals, whereas coeloconic sensilla perform additional chemosensory roles.^28^ A single sensillum may house multiple OSNs, each responsive to different compounds, enabling complex odor discrimination.^27^ Before odorants can activate the ORs, they must be solubilized and transported across the hydrophilic sensillar lymph. This transport process, termed “peri-receptor events” in vertebrate olfaction, involves an ensemble of protein families including OBPs that act as molecular chaperones.^29,30^ This orchestrated transport and transduction sequence underpins the insect’s ability to detect and respond to complex odor landscapes.

### 1.2 Odorant-Binding Proteins (OBPs)

OBPs are a large family of small (generally 10–20 kDa), acidic, water-soluble extracellular proteins that bind and transport hydrophobic odorants, including pheromones, across the aqueous sensillar lymph to ORs.^31,32^ Initially identified in 1981 by Vogt and Riddiford, OBPs belong to the superfamily “lipocalins” and were first characterized through the discovery of *Antheraea polyphemus* pheromone-binding protein (ApolPBP1).^33^ OBPs exhibit a cysteine-rich structure and are present in high concentrations (up to 10 mM), uniformly distributed within the sensillar lymph.^33^ Their function ensures that volatile hydrophobic molecules, which would otherwise remain in the atmosphere, can cross the aqueous lymph to reach sensory neurons and initiate signal transduction cascades.^30,31^

In lepidopteran insects, OBPs are categorized into subfamilies based on sequence homology and functional characteristics.^34^ These include (i) pheromone-binding proteins (PBPs), (ii) general odorant-binding proteins (GOBP1 and GOBP2), and (iii) antennal binding proteins (ABP1 and ABP2).^34^ Full genome examinations across major insect orders suggest that the number of Classic-OBP genes can range from 10 to 50 per species.^35^ In Lepidoptera specifically, the number typically ranges from 19 to 29 genes.^35,36^ Among these, GOBPs are involved in the detection of plant volatiles and other general odorants, whereas PBPs represent evolutionary adaptations of OBPs to species-specific pheromones.^37^ However, some studies indicate that GOBPs, particularly in larval stages, may also be tuned to sex pheromones, suggesting a broader functional role than previously assumed.^38–40^ Furthermore, PBPs are primarily expressed in males and are critical for sexual communication, while GOBPs are expressed in both sexes.^37^ OBPs are further classified into structural subclasses based on the number and position of cysteine residues^41,42^ (Table 1).

**Table 1:** Classification of Odorant-Binding Proteins by Conserved Cysteine Residues

name of OBP	number of conserved cysteines	examples
classic OBPs	6 cysteine residues (6C), subdivided by chain and C-terminus length	*Bombyx mori*,^123^ *Antheraea polyphemus* ^123^ (long chain)
	long-chain OBPs – 140–160 amino acids	*Apis mellifera* ^123^ (medium chain)
	medium-chain OBPs – ∼120 amino acids	*Leucophaea maderae* ^123^ (short chain)
	short-chain OBPs – 100–110 amino acids	
plus-C OBPs	6 cysteine residues + 2 additional cysteine residues (8C) + conserved proline	*Drosophila* OBP99a,^42^ *Anopheles gambiae* OBP5470,^42^ *Aedes aegypti* OBP83A,^42^ *Bombyx mori* OBP 41 and 43 ^178^
minus-C OBPs	<6 cysteine residues, usually 4 cysteine residues (4C)	*Acyrthosiphon pisum*,^41^ *Helicoverpa armigera* OBP17 and 18,^250^ *Monochamus alternatus* OBP1,^251^ *Helicoverpa armigera* OBP 18,^178^ dipteran insects
atypical OBPs	6 cysteines + 1 additional cysteine at C terminus	*Anopheles gambiae* OBP35,^41^ *Aedes aegypti*,^41,252^ *Culex quinquefasciatus* ^253^
C8 OBPs	8 cysteine residues (8C) with 4 disulfide bonds + extended C terminus	*Anopheles gambiae* OBP7 ^254^

### 1.3 Insect Olfactory Receptor Complexes and Signal Transduction

Insect chemoreception relies on diverse receptor families, including large repertoires of ORs, a smaller number of gustatory receptors (GRs), and ionotropic receptors (IRs), expressed in the dendritic membranes of OSNs.^43^ Each OSN typically expresses a single receptor type, adhering to the “one receptor per neuron” rule, enabling precise detection of odorants and pheromones, especially for species-specific mating and territorial cues.^11,44^ ORs are primarily localized in sensory appendages such as antennae and maxillary palps.^45^ First identified in the fruit fly *Drosophila melanogaster* via antennal cDNA sequencing, ORs have since been characterized across many moth species, including *Bombyx mori* (silkworm moth), *Manduca sexta* (tobacco hornworm), *Spodoptera litura* (oriental leafworm), *Heliothis virescens* (tobacco budworm), *Helicoverpa assulta* (oriental tobacco budworm), *Helicoverpa armigera* (cotton bollworm), *Chilo suppressalis* (striped rice borer), *Operophtera brumata* (winter moth), *Epiphyas postvittana* (light brown apple moth), *Ostrinia furnacalis* (Asian corn borer), *Planotortrix octo* and *Planotortrix excessana* (greenheaded leafrollers), *Conogethes punctiferalis* (yellow peach moth), and *Eriocrania semipurpurella* (purplish birch-miner moth).^45–53^

Insect ORs were initially assumed to belong to the G-protein-coupled receptor (GPCR) family, based on their characteristic seven-transmembrane (7-TM) domain.^54^ However, it is now well established that insect ORs and GRs exhibit an inverted membrane topology compared to classical vertebrate GPCRs, with the N-termini oriented intracellularly, suggesting a signaling mechanism distinct from GPCRs.^54^ Indeed, insect ORs operate as heteromeric complexes, comprising a variable (odorant-specific OrX or a pheromone-specific OrY) subunit with the odorant-binding site paired with a highly conserved coreceptor, Orco.^55^ Identified in *D. melanogaster* as Or83b, Orco shares the reversed membrane topology characteristic of insect ORs, facilitates their localization to the dendritic membrane, and forms ligand-gated cation channels critical for sensory neuron activation.^54,56–58^ Far from being a passive partner, the Orco plays a critical role in olfactory signaling. Orco–/– mutants in *H. armigera* and other moth species exhibit pronounced defects in pheromone detection, host-seeking behavior, and reproduction, including male infertility and loss of oviposition selectivity in females.^59–61^

The olfactory transduction cascade is initiated when odorants, including pheromones, bind to specific OBPs in the sensillar lymph.^62^ This ligand-OBP complex subsequently interacts with ORs, potentially aided by sensory neuron membrane proteins (SNMPs).^63^ SNMPs, homologous to the vertebrate transmembrane protein family CD36, were originally identified in *A. polyphemus* trichoid sensilla and are now known to be broadly conserved and functionally critical across insect taxa.^64–66^ Localized to OSN dendritic membranes alongside ORs, they mediate hydrophobic ligand transfer from OBPs to ORs and aid in signal termination post activation.^67^

Ligand binding to the OR-Orco complex induces conformational changes that trigger signal transduction via one of two proposed mechanisms^58,68^ (Figure 2). The ionotropic model where OR-Orco complexes function as ligand-gated ion channels, directly depolarizing OSNs, represents the primary and widely accepted mechanism of insect olfactory transduction.^8,13,58,62,68–70^ Alternatively, a metabotropic model observed in certain moth species proposes that odorant binding activates G-protein signaling, which in turn stimulates phospholipase C (PLC)-mediated hydrolysis of phosphatidylinositol 4,5-bisphosphate (PIP2) into inositol 1,4,5-trisphosphate (IP3) and diacylglycerol (DAG).^8,13,62,68–71^ These secondary messengers modulate intracellular calcium and ion channel activity, respectively.^8,13,68–71^ Though cyclic nucleotides (e.g., cAMP and cGMP) have been proposed as additional second messengers, their functional relevance remains unclear, and electrophysiological studies support a minimal role for metabotropic pathways in general under physiological conditions.^8,69,71^

**Figure 2:**
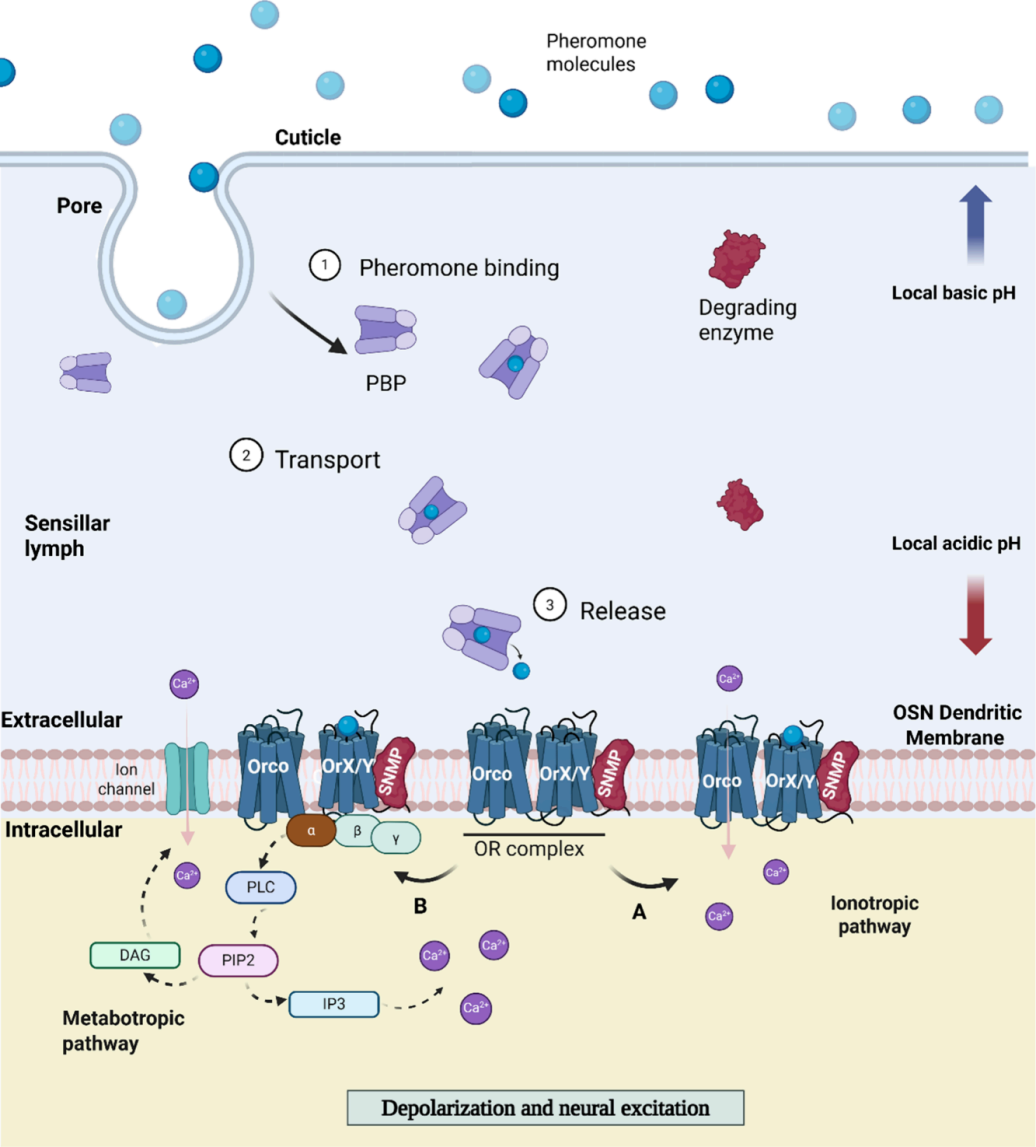
Schematic representation of sensillar environment, pheromone transport and odorant detection pathways. Key components include olfactory receptors (ORs), coreceptors (Orco), sensory neuron membrane proteins (SNMP), odorant sensory neurons (OSNs), pheromone-degrading enzymes (PDEs) and pheromone-binding proteins (PBPs). After transport through the sensillar lymph, the pheromone–PBP complex binds to the OrX/Y–Orco receptor complex, activating ionotropic (A) or metabotropic (B) signaling pathways. The ionotropic (A) pathway directly allows calcium influx; the metabotropic pathway (A) involves G-protein signaling, activating phospholipase C (PLC), which hydrolyzes PIP2 into DAG and IP3, leading to further calcium release. However, the role of the metabotropic pathway in insect olfactory transduction remains debated, with current evidence suggesting it plays a relatively minor role. The schematic reflects both proposed mechanisms. Created in BioRender in collaboration with Smita Mohanty. Ray, I. (2025) https://BioRender.com/8g34e6m.

Following receptor activation, pheromones are inactivated either enzymatically or through sequestration by molecular traps.^22,24–26,35^ Among the enzymatic mechanisms, pheromone-degrading enzymes (PDEs), particularly antennal carboxylesterases (CXEs), play a critical role in rapidly clearing odorants from the olfactory environment.^24,25,72^ In *S. litura* and *S. exigua*, several well-documented PDEs such as SlCXE7 and SexiCXE11 have been shown to efficiently hydrolyze acetate pheromone components, ensuring high temporal resolution of olfactory signaling.^22,25,73^ These PDEs exhibit tissue-specific expression and substrate selectivity, reflecting evolutionary adaptation to species-specific pheromone blends.^25,72,73^ Their rapid degradation activity not only prevents signal saturation but also primes olfactory sensory neurons for subsequent stimulation, underscoring their essential role in pheromone signal termination.^74^ The functional interplay among ORs, Orco, SNMPs, and OBPs remains an active area of research. Key interactions relevant to pheromone perception will be further discussed in Section 6.

### 1.4 Pheromones

Given the central role of these proteins in pheromone detection, it is important to consider the nature and classification of pheromones themselves. The term “pheromone,” derived from the Greek words “pherein” (to carry) and “hormo̅n” (to excite), was introduced by Peter Karlson and Martin Lüscher in 1959.^75^ Functionally, pheromones are classified into two primary types: releaser pheromones, which evoke immediate behavioral responses such as mate attraction, aggregation, or alarm signaling, and primer pheromones, which elicit prolonged physiological changes influencing behaviors or developmental processes, particularly in eusocial insects.^76^

Sex pheromones, predominantly synthesized by females in minute quantities, serve as attractant compounds in males that signal the presence and reproductive maturity of potential mates.^4^ Additionally, male moths respond exclusively to sex pheromone signals emitted by conspecific females, demonstrating the ability to distinguish self-from nonself-chemical cues.^4^ These pheromones are further categorized into sex attractants, which induce long-distance, upwind orientation toward conspecifics, and courtship pheromones, which trigger close-range, mating-related behaviors.^77^ The structural elucidation of the first sex pheromone, (*E,Z*)-10,12-hexadecadienol (bombykol) from *B. mori* in 1959, marked the beginning of extensive research on lepidopteran pheromones, with over 600 species-specific pheromones characterized to date.^78^ Lepidopteran sex attractant pheromones represent the most extensively studied class of releaser pheromones, functioning as critical modulators during male flight orientation toward females.^32^

Structurally, pheromones are diverse. Typically, they are C10–C18 straight-chain unsaturated compounds with oxygenated functional groups encompassing hydrocarbons, alcohols, esters, epoxides, aldehydes, ketones, lactones, carboxylic acids, isoprenoids, and triacyl glycerides.^79^ Based on production, chemical structure, and biosynthetic origin, pheromones have been classified into four main types (Tables 2 and[Table tbl3]).^80,81^ The term “pheromone blend” is used as insects often employ a unique combination of chemicals in precise ratios, which, coupled with the structural diversity of pheromones, contributes to their species-specific functionality.^79^ For instance, the wild silk moth *A. polyphemus* utilizes a sex pheromone blend consisting of approximately 90% (*E,Z*)-6,11-hexadecadien-1-yl acetate and 10% (*E,Z*)-6,11-hexadecadienal.^82^

**Table 2:** Classification of Pheromones Based on Chemical Structure and Biosynthetic Origin

**pheromone type**	characteristics	examples
Type I	C10–C18 monounsaturated or diunsaturated acetates, alcohols, and aldehydes^81,255^	∼75% of known moth pheromones^255^
Type II	polyunsaturated straight-chain hydrocarbons and epoxide derivatives with C17–C23 carbon atoms^81,256^	∼15% of identified moth pheromones^256^
Type III	functionalized saturated/unsaturated hydrocarbons containing methyl branches^81,257^	
Type 0	short-chain methyl carbinols and methyl ketones^80,81^	found in primitive moth species (*Eriocraniidae*, *Trichoptera*)^80^
unclassified pheromones	propionate esters, methyl-branched alcohols, (Z)-7-alken-11-ones^80^	have distinct biosynthetic origins and do not fit within the four main types^80^

**Table 3:**
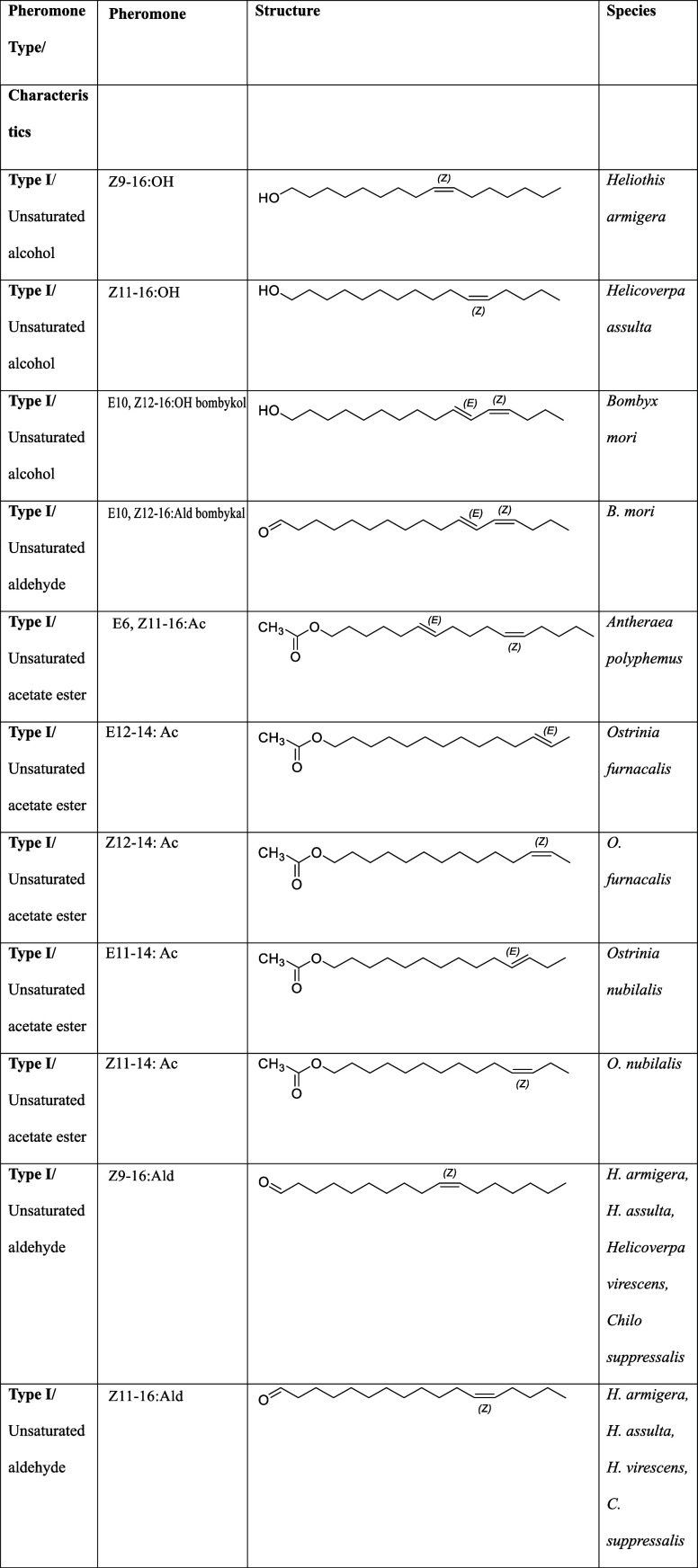

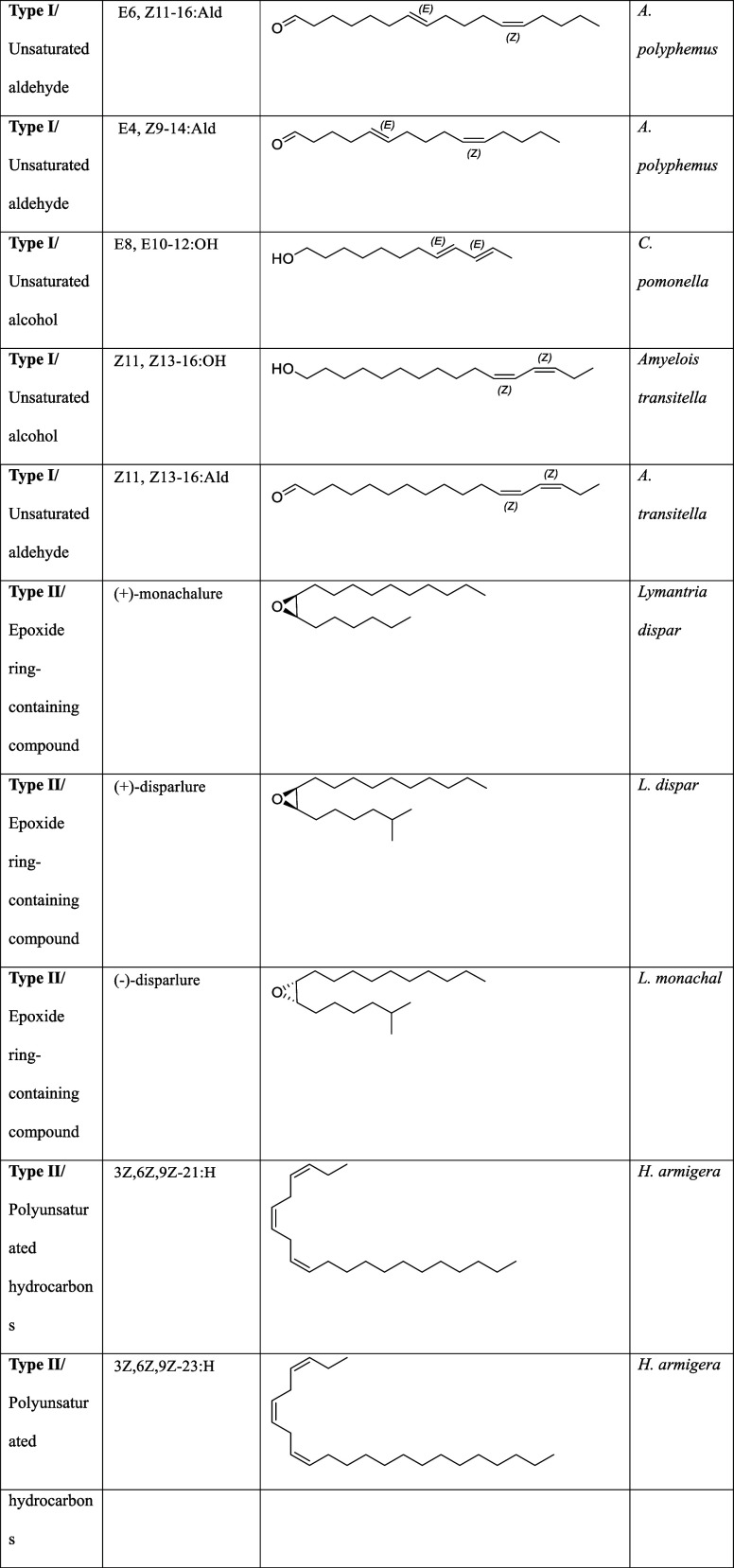
Chemical Structures of Various Pheromones^a^

a Structures created using ChemDraw, Version 23.1.

Similarly, the chirality of these compounds is critical for their function. The gypsy moth, *Lymantria dispar*, uses (7*R*,8*S*)-cis-2-methyl-7,8-epoxyoctadecane, also known as (+)-disparlure, as the major component of its sex pheromone.^83^ Interestingly, a closely related species, the nun moth (*L. monachal*), relies on a mixture of (±)-disparlure and (±)-monachalure (7,8-epoxyoctadecane) as its sex pheromone.^83^ In this instance, (−)-disparlure is neither attractive nor repellent to gypsy moth males on its own, but when presented alongside (+)-disparlure, it disrupts upwind flight behavior, effectively neutralizing the response to the pheromone.^84^ While most moths rely on either Type I or Type II pheromones, some species, such as *Hyphantria cunea* (the fall webworm) and *Orthaga achatina*, employ a combination of both.^85,86^ For instance, for *H. cunea*, the pheromone blend includes Type I aldehydes (e.g., *Z*9, *Z*12–18: Ald) and Type II polyenes (e.g., Z3, Z6–9*S*, 10*R*-epoxy-21Hy and 1, *Z*3, *Z*6–9*S*, 10*R*-epoxy-21Hy), presenting a unique challenge for PBPs, which must efficiently bind and transport structurally distinct ligands to their corresponding ORs.^86^

However, the hydrophobic nature of pheromones, which is essential for their rapid dispersion in air, presents a challenge to their transport through the aqueous sensillar lymph that surrounds the OSNs. Transport across this hydrophilic barrier is facilitated by OBPs, particularly PBPs, which solubilize and deliver pheromones to ORs, enabling effective chemosensory signal transduction.^32^

### 1.5 Moth Pheromone-Binding Proteins

In lepidopteran male moths, pheromone reception is a highly dynamic process facilitated by an olfactory system that is finely tuned for the detection of female-emitted sex pheromones. A key component of this system is the family of pheromone-binding proteins (PBPs), which are small, acidic, and highly soluble extracellular proteins essential for peripheral pheromone detection.^5^ PBPs transport hydrophobic pheromone molecules through the aqueous sensillar lymph to the ORs, thereby playing a critical role in the initial stages of signal transduction. These proteins are synthesized by two types of olfactory accessory cells, trichogen and tormogen cells, and are highly concentrated in the sensillar lymph (∼10 mM) of male moth antennae.^87–90^ PBPs solubilize the otherwise hydrophobic pheromone molecules and facilitate their delivery to ORs located on the dendritic membranes of olfactory sensory neurons (OSNs)^27,28^ (Figure 2). This process ensures efficient pheromone capture and receptor activation, contributing to the sensitivity and specificity of the moth’s olfactory system on the dendritic membranes of OSNs^32,33^ (Figure 2). The high concentration of PBPs in the sensillar lymph ensures that virtually every molecule of pheromone efficiently binds to a carrier protein.^5^ This binding is critical, as the transport of hydrophobic pheromone molecules through the aqueous antennal lymph to their respective receptors may represent the rate-limiting step in pheromone detection in moths and other insect species.

The abundance of PBPs suggests that, beyond their role in transport, they may contribute to multiple aspects of pheromone perception. These include facilitating pheromone selectivity and recognition, pheromone concentration in the hydrophilic lymph, promoting desorption of pheromones from the cuticular wax layer into the aqueous environment, and scavenging residual pheromones from the surrounding environment to maintain signal fidelity.^6,91–93^ Additionally, they provide protection from enzymatic degradation by encapsulating pheromones within hydrophobic binding pockets, prevent receptor saturation, and potentially serve as cofactors in the activation of ORs.^74,91,92^ Many moth species express multiple PBPs, each exhibiting distinct binding affinities for specific pheromone components, as demonstrated by *in vitro* binding assays.^85^ This molecular specificity is reported to be essential for species-specific chemical communication.^94^ Structural analyses, molecular docking simulations, and *in vitro* binding studies have provided important insights into the functional mechanisms of PBPs. These investigations have identified a central hydrophobic binding pocket and have revealed specific amino acid residues critical for the binding interaction with a sex pheromone molecule.

The first PBP was identified in the male antennal tissues of the *A. polyphemus* using a radio-labeled (^3^H) pheromone for their role in pheromone detection.^33^ This protein was also the first to be successfully cloned and heterologously expressed in bacterial systems.^95,96^ Since the initial discovery, orthologs have been identified in over 30 lepidopteran species through homologous cloning and transcriptomic analyses.^97^ Early studies primarily focused on isolating these proteins from insect antennae and characterizing them as a class of passive carrier proteins. In addition to *A. polyphemus*, PBPs from species such as *B. mori*, *L. dispar*, *H. assulta*, and *Plutella xylostella* have been extensively studied. These species have served as model systems for elucidating ligand-binding specificity, protein structure, and the molecular mechanisms underlying pheromone detection.^98–100^

## 2 Structural Studies on Lepidopteran Pheromone-Binding Proteins

The first high-resolution structure of an invertebrate PBP was determined for the silk moth *B. mori* (BmorPBP, later renamed BmorPBP1 following the identification of multiple PBPs in the species^101^) in complex with its primary sex attractant pheromone, bombykol ((*E, Z*)-10,12-hexadecadien-1-ol).^37^ This structure was solved by single-crystal X-ray diffraction at 1.8 Å resolution.^102^ Female *B. mori* releases a blend of sex pheromones consisting primarily of bombykol and a minor component, bombykal, (*E, Z*)-10,12-hexadecadienal), typically in an 11:1 ratio.^103^ While bombykol alone is sufficient to elicit behavioral response in male moths, bombykal acts as a negative modulator, suppressing the initiation of this behavior.^104,105^

### 2.1 Primary Structure and Sequence Conservation

Moth PBPs typically consist of 130–150 amino acids, ranging from 13 to 20 kDa in size.^33^ Multiple PBP paralogs are often expressed within a single lepidopteran species, enabling the simultaneous detection of various semiochemicals.^94^ In general, noctuid species express three to four distinct PBPs, whereas non-noctuid species may express even more.^94^ This functional diversity is advantageous as many species rely on a complex blend of semiochemicals rather than a single compound for effective pheromone signaling.

PBPs from different moth species generally share about 50% sequence identity, whereas sequence identity between PBPs and GOBPs is lower, at 30%.^106–108^ Sequence alignment analyses have revealed a six strictly conserved cysteine signature that forms three disulfide bonds, which are crucial for maintaining protein stability and the overall three-dimensional structure.^109^ Additionally, three histidine residues (His69, His70, and His95) are also conserved across most lepidopteran PBPs.^37^

Despite sharing a common structural framework, variations in specific amino acid residues influence substrate specificity and contribute to species-specific pheromone recognition. For instance, *Ostrinia nubilalis* PBP3 (OnubPBP3) shares over 50% sequence identity with well-characterized lepidopteran PBPs, including BmorPBP1, ApolPBP1, *Amyelois transitella* PBP1 (AtraPBP1), and *Lymantria dispar* PBP2 (LdisPBP2).^108^ However, OnubPBP3 displays notable differences in two key structural regions: the histidine gate, where the conserved His70 is substituted by Arg70, and a C-terminal gate (Pro128-Ser144), which is more polar due to the presence of four additional charged residues.^108^ Similar structural variations are observed in other PBPs, such as OfurPBP2 from *Ostrinia furnacalis*. This protein features a unique His88 residue within the α5 helix, a characteristic absent in other PBPs.^110^ This sequence-level plasticity within a conserved fold is a hallmark of protein families under adaptive pressure. In PBPs, it allows evolutionary fine-tuning of ligand-binding specificity while preserving the structural chassis.

These structural variations enhance the specificity and adaptability of PBPs, allowing for precise pheromone discrimination across diverse ecological and evolutionary contexts. At the same time, the high sequence homology and functional similarities observed between PBPs from different species facilitate comparative studies to understand the roles of conserved amino acid residues. For example, LdisPBP2 shares up to 76% sequence identity with ApolPBP1, despite the fact that the two species detect distinct pheromones.^111^ Sequence homology has enabled the use of experimentally determined PBP structures as templates for predicting the structures of newly identified PBPs by using computational modeling. For example, Yu et al. used ApolPBP1 as a template to model LdisPBP2, while EposPBP3 was used to generate the model of TabsPBP3.^111,112^ These studies highlight the effectiveness of homology-based modeling in advancing structural insights into PBPs across diverse lepidopteran species.

### 2.2 Secondary and Tertiary Structure

The first X-ray crystal structure of BmorPBP1 in complex with bombykol, reported by Sandler et al., sparked significant interest in understanding the structural basis of PBP function.^37^ To date, approximately ten X-ray crystal structures and nine NMR structures of lepidopteran PBPs have been resolved and deposited in the Protein Data Bank (PDB) database (https://www.rcsb.org/pdb/home/home.do), providing valuable insights into their secondary and tertiary structural features (Table 4).

**Table 4:** Structural Studies of Pheromone-Binding Proteins (PBPs) in Various Moth Species

species	number of X-ray structures	number of NMR structures	total structures	PDB IDs
*Bombyx mori* (Silkworm)	4	3	7	X-ray: 1DQE,^37^ 2FJY,^258^ 2P70,^98^ 2P71 ^98^
				NMR: 1GM0,^259^ 1LS8,^260^ 1XFR^261^
*Antheraea polyphemus*	0	3	3	NMR: 1QWV,^116^ 1TWO,^118^ 2JPO^262^
*Amyelois transitella*	2	1	3	X-ray: 4INW,^93^ 4INX^93^
				NMR: 2KPH^115^
*Helicoverpa armigera*	3	0	3	X-ray: 7VW9,^119^ 7VW8,^119^ 7VWA^119^
*Lymantria dispar*	0	1	1	NMR: 6UM9 ^117^
*Epiphyas postvittana*	1	0	1	X-ray: 6VQ5 ^120^
*Ostrinia furnacalis*	0	1	1	NMR: 7UO6 ^110^

Unlike vertebrate OBPs, insect PBPs are helical proteins typically composed of six to seven α-helices connected by flexible loops, forming a compact globular structure.^37,113,114^ The protein’s hydrophobic binding pocket, crucial for pheromone capture, is nestled between these helices, providing a stable environment. Four antiparallel helices, α1, α4, α5, and α6, form the core of the pocket and converge at the narrow end, while helix α3 caps the opposite end of the pocket^37,115,116^ (Figure 3). Helix α3 is stabilized by Cys19–Cys54 and Cys50–Cys108 disulfide bonds, which anchor it to the adjacent α1 and α6 helices. A third disulfide bond, Cys97 and Cys117, bridges helices α5 and α6, further contributing to the overall structural stability of the protein.^37,115^ This disulfide-bonded architecture is critical for both the stability and function of PBPs. PBPs undergo conformational changes in response to environmental factors, such as pH and hydrophobic ligands. At neutral pH, the hydrophobic pocket tightly holds the pheromone for efficient transport. In acidic conditions, such as those near the OSNs, a conformational switch destabilizes the ligand-protein interaction, releasing the pheromone for receptor activation. While variations in sequence and structure exist, the fundamental ability to capture, transport, and release pheromones remains a shared characteristic in many organisms, underscoring the critical role of PBPs in chemical communication.

**Figure 3:**
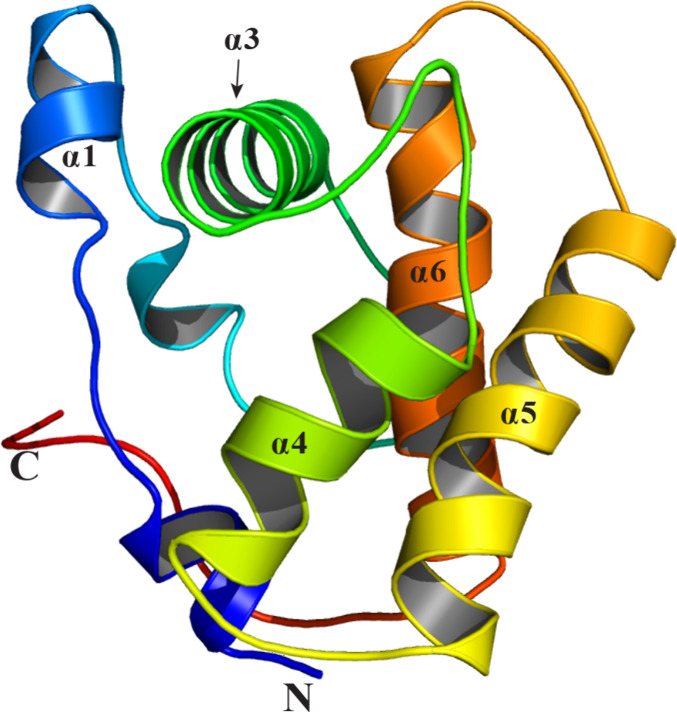
Representative structural organization of the pheromone-binding pocket in moth PBPs. The hydrophobic pocket is formed by four core helices (α1, α4, α5, and α6) converging at the narrow end, while helix α3 caps the opposite end.

### 2.3 Ligand Binding Mechanisms and Structural Determinants of Pheromone Recognition

Elucidating the ligand-binding mechanisms of PBPs is fundamental to understanding the molecular basis of specificity and sensitivity in insect pheromone perception. As a result, numerous studies have focused on the *in vitro* binding analysis of moth PBPs.^110^ ,^117^ −^118,119^ Key binding interactions within the hydrophobic binding pocket—primarily hydrophobic forces, van der Waals interactions, and occasionally hydrogen bonds—govern the selectivity and efficiency of pheromone transport.^108,120–123^ These interactions occur between conserved residues lining the pocket and the ligand.^20,91,123–126^ Mutagenesis approaches, including site-directed and alanine-scanning mutagenesis, have identified critical residues that influence the binding affinity and specificity. For example, substituting specific amino acids helps understand how changes in polarity, charge, or steric hindrance impact affinity and selectivity, while systematic alanine substitutions help pinpoint structurally important positions. These studies underscore how subtle changes in the PBP structure can accommodate chemically diverse pheromones, revealing functional diversity among lepidopteran species.

#### 2.3.1 pH-Dependent Binding and Release Mechanism

Biochemical, structural, and binding studies of a few lepidopteran PBPs, including ApolPBP1, AtraPBP1, BmorPBP1, and LdisPBPs, have revealed a conserved, pH-dependent mechanism that regulates ligand binding and release. These proteins bind ligands at neutral to basic pH (≥6.0), corresponding to the pH of the sensillar lymph, and release them at acidic pH (∼4.5), typically found near the membrane-bound receptors^108,110,117,127,128^ (Figure 4). This process involves a pH-dependent conformational switch, primarily regulated by two biological gating mechanisms: (i) the histidine gate, consisting of His70 and His95, and (ii) the C-terminal gate involving ∼14–16 terminal residues^108,110,117,127,128^ (Figure 4). Their cooperative mechanism, involving pH-dependent conformational changes that regulate the opening and closing of the binding pocket, ensures that ligand release is precisely coordinated with the spatial and temporal dynamics of OR activation. This mechanism minimizes signal degradation and is widely conserved among lepidopteran PBPs, while it still allows for species-specific functional adaptations.

**Figure 4:**
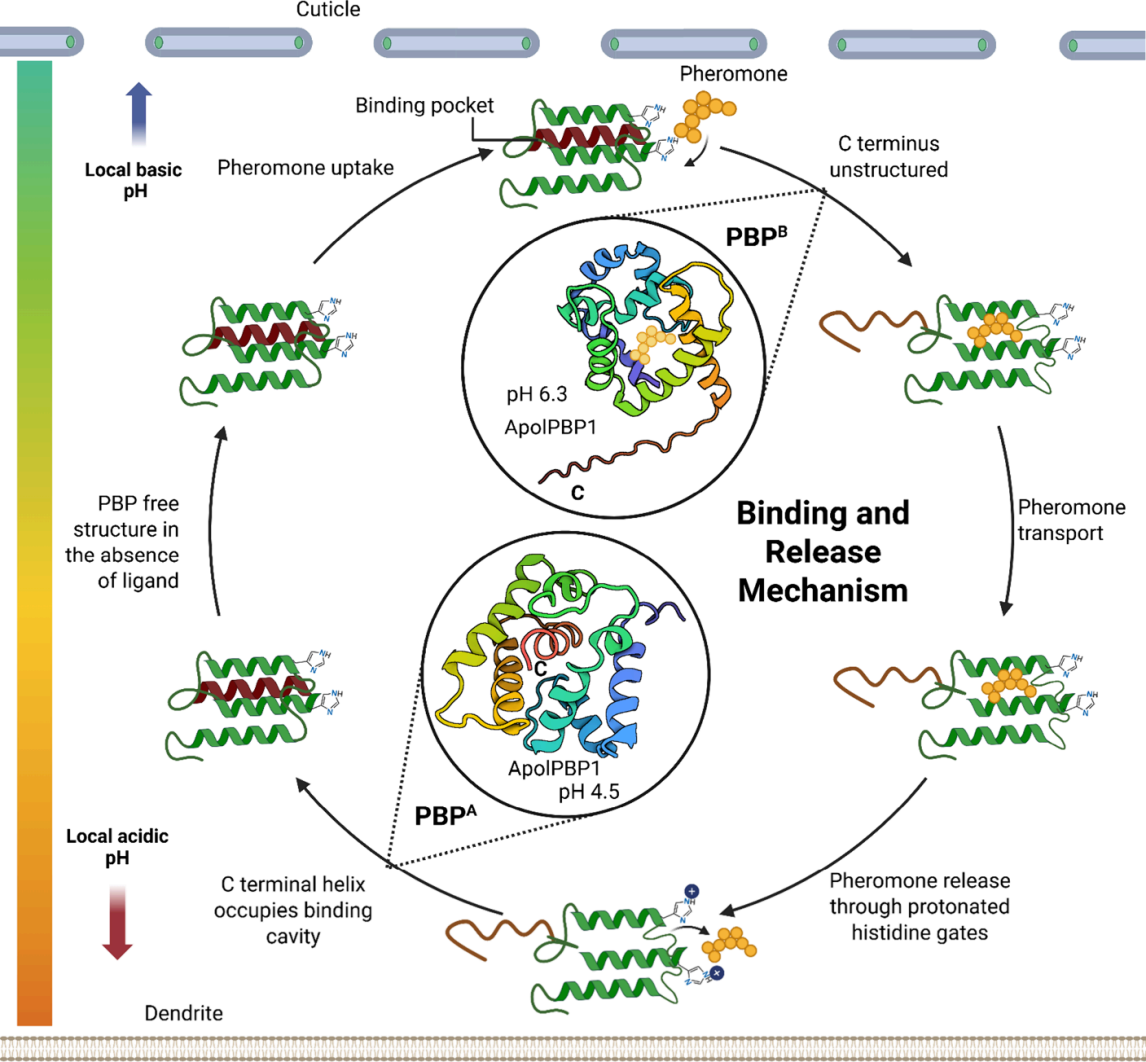
Schematic representation of pheromone binding and release mechanism by pheromone-binding proteins (PBPs). Representative pH-Induced Conformational Changes shown within the schema using ApolPBP1 structures: the open conformation at pH 4.5 (PDB: 2JPO) facilitates ligand release, while the closed conformation at pH 6.5 (PDB: 1QWV) enables ligand binding. The C-terminus in red in both states. Created in BioRender in collaboration with Smita Mohanty. Ray, I. (2025) https://BioRender.com/65itn35.

The accepted ligand binding and release mechanism of PBPs involves a switch between two major conformations, PBP^A^ and PBP^B^, regulated by the pH and presence of the ligand (Figure 4). Both share a conserved structural framework of six core α-helices (α1−α6), stabilized by three strictly conserved disulfide bridges: Cys19-Cys54 links helices α1 and α3, Cys50-Cys108 bridges helices α3 and α6, and Cys97-Cys117 connects helices α5 and α6.^110^ In contrast, the N-terminal and C-terminal segments of the protein adopt either a helical or extended structure depending on pH and the occupancy of the ligand in the binding pocket.^110^ This structural flexibility enables the conformational switch that is essential for ligand binding at neutral to basic pH and its release under acidic conditions.

In the PBP^A^ or apo (acidic) conformation at pH 4.5, the C-terminus is tucked inside the hydrophobic pocket as a structured α-helix (α7), effectively preventing ligand access, while the N-terminus remains disordered (Figure 5). The C-terminal α7 helix is a dodecapeptide predominantly composed of hydrophobic residues and three highly conserved acidic residues, arranged in a way that forms two hydrophobic faces.^127^ The larger hydrophobic face is oriented toward the broader internal binding pocket, while the smaller face aligns antiparallel to α4 and interacts with it.^127^ All A-form structures share additional conserved features, including a kinked helix α3 and two nonregular β-turn motifs characterized by i → i+3 backbone hydrogen bonding.^127^ These β-turn motifs are located in the loop region connecting helices α3 and α4.^127^ Consequently, helix α3 is designated into two distinct segments: α3a, comprising residues preceding the kinked residue, and α3b, comprising residues following it.^127^ Up to a 10-fold decrease in ligand affinity is observed under acidic conditions relative to neutral pH.^92,124,129,130^ This “autoinhibitory” mechanism ensures that ligand release occurs in the immediate vicinity of ORs.

**Figure 5:**
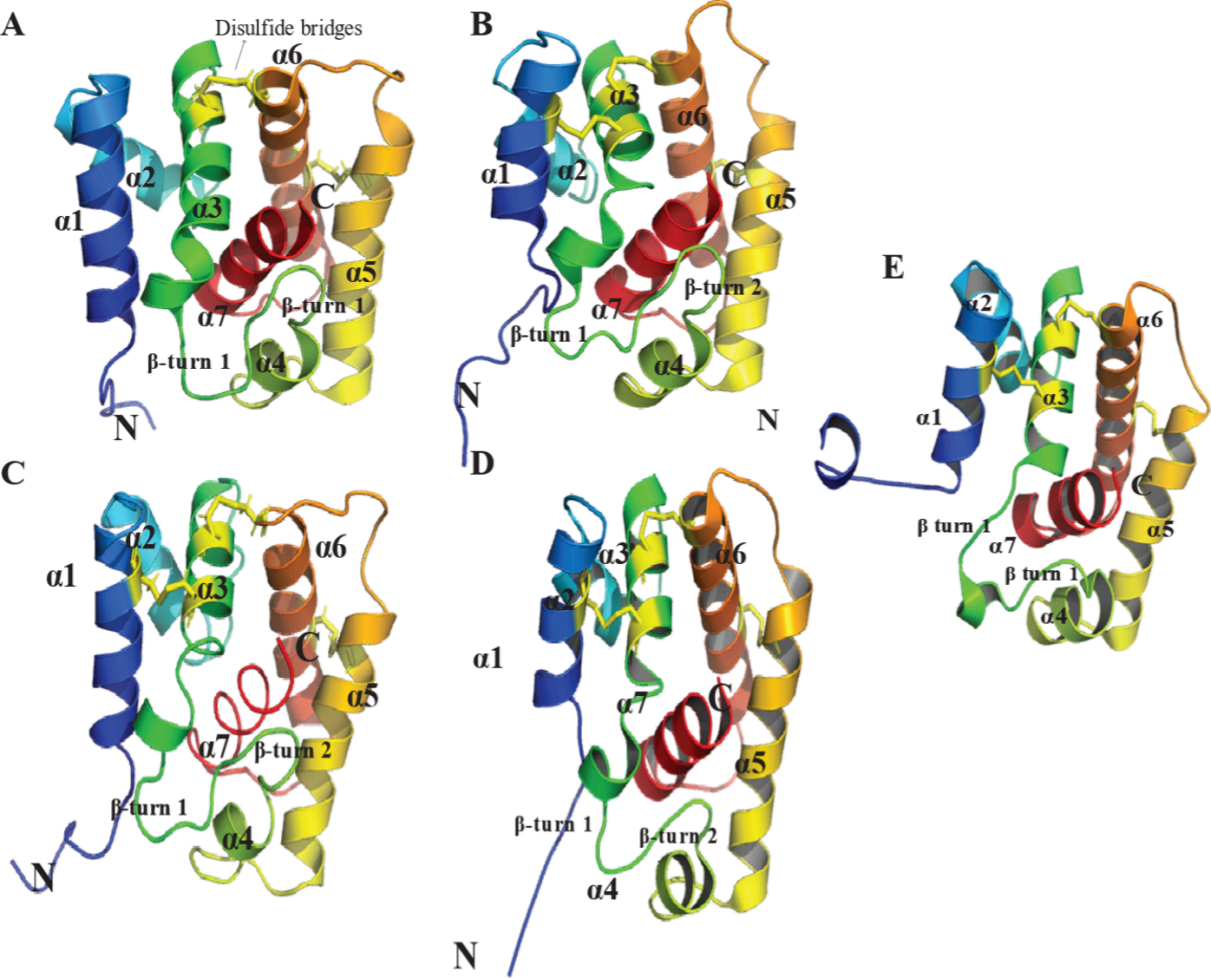
Structural Comparison of Moth PBP A-Form Conformations. This figure illustrates the A-form structures of moth PBPs, labeling helices α1-α7 and β-turn motifs. Conserved disulfide linkages are shown as sticks in yellow. (A) BmorPBP1 (PDB ID: 1GM0), averaged NMR structure at pH 4.5, (B) BmorPBP1 (PDB ID: 2FJY), X-ray structure at 2.30 Å resolution, delipidated, pH 7.5, (C) ApolPBP1 (PDB ID: 2JPO), averaged NMR structure at pH 4.5, (D) AtraPBP1 (PDB ID: 2KPH), lowest energy NMR structure at pH 4.5, (E) LdisPBP1 (PDB ID: 6UM9), averaged NMR structure at pH 4.5. Structural visualization was performed using PyMOL.^249^

In the PBP^B^ or holo (basic) conformation (Figure 6), the C-terminus adopts an elongated, random-coil conformation that extends outside the hydrophobic pocket exposed to the solvent. In this state, the N-terminus forms an additional helix.^127^ This conformational change occurs at higher pH, typically near the cuticular pores, and in the presence of pheromonal or nonpheromonal ligands. The switch to this open conformation facilitates the binding of ligands and prepares the protein for ligand transport. Several PBP^B^ structures containing ligands endogenous to the bacterial expression systems have been documented.^128,131^ These endogenous ligands mimic pheromones, triggering a conformational switch to the PBP^B^ form at pH levels above 6.0.^128^ This transition allows the protein to adopt the holo or open conformation, facilitating ligand binding and mimicking the behavior of PBPs in their natural physiological context.^128^ The helical N-terminus of holoPBP, designated as helix α1a, contributes to stabilizing this state. Helix α4 is elongated, incorporating residues from the loop connecting α3 and α4, reflecting a change in loop dynamics associated with ligand binding.^127^

**Figure 6:**
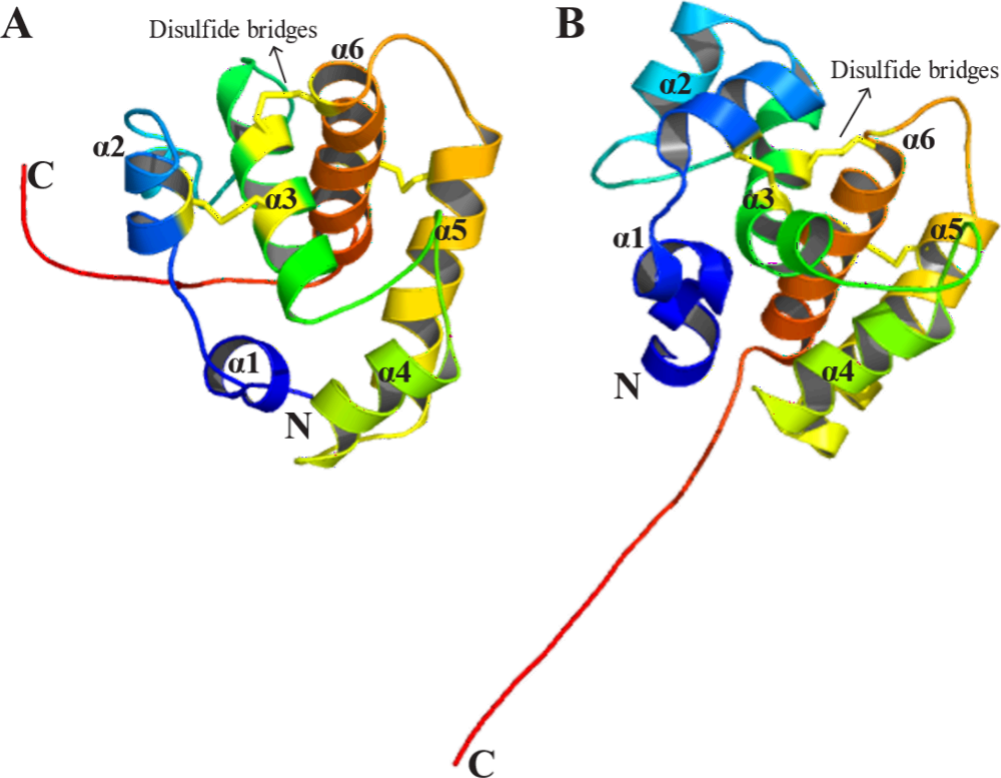
NMR Structures of Moth PBPs in the B-Form: (A) BmorPBP1 (PDB ID: 1LS8, pH 6.5), (B) ApolPBP1 (PDB ID: 1QWV, pH 6.3). Structural visualization was performed using PyMOL.^249^

#### 2.3.2 Structural Basis of Conformational Switching

The pH-dependent conformational switching between the holo (ligand-bound, basic or PBP^B^) and apo (ligand-free, acidic or PBP^A^) conformations of several PBPs has been well-characterized through biochemical and biophysical methods, including circular dichroism (CD) and heteronuclear two-dimensional (2D) NMR.^115,117,128,132–135^ The 2D ^1^H–^15^N-HSQC NMR spectrum provides a high-resolution structural fingerprint of a protein and is widely used to monitor conformational changes in response to pH, temperature, and ligand presence. In ApolPBP1, NMR titration studies have revealed that ligand binding is essential for the pH-driven transition between the closed (PBP^A^) and open (PBP^B^) conformations.^128^ The apo and holo ApolPBP1 show dramatically different responses to pH changes under identical conditions: holo ApolPBP1 switches its conformation from PBP^B^ to PBP^A^ when the pH drops from above 6.0 to below 5.0, whereas apo ApolPBP1 remains in the PBP^A^ conformation across all tested pH levels.^128^ These titration data also demonstrate the transition from PBP^A^ to PBP^B^ conformation is both ligand-dependent and pH-sensitive, occurring only when the pH is ≥6.0 and at a 1:1 protein-to-ligand ratio—underscoring the critical role of ligand occupancy in enabling this structural transition.^128^

The roles of the histidine gate (His70 and His95) and the C-terminal gate were elucidated through mutagenesis and binding assays (Figures 7 and 8). Substituting His70 and His95 with alanine shifts the equilibrium toward the PBP^B^ conformation, even under acidic conditions, indicating that these residues are essential for protonation-triggered gating.^116^ Notably, the histidine gate is implicated mainly in ligand release rather than binding, as mutants (such as ApolPBP1H70A/H95A) do not have altered affinities toward the ligand.^116^

**Figure 7:**
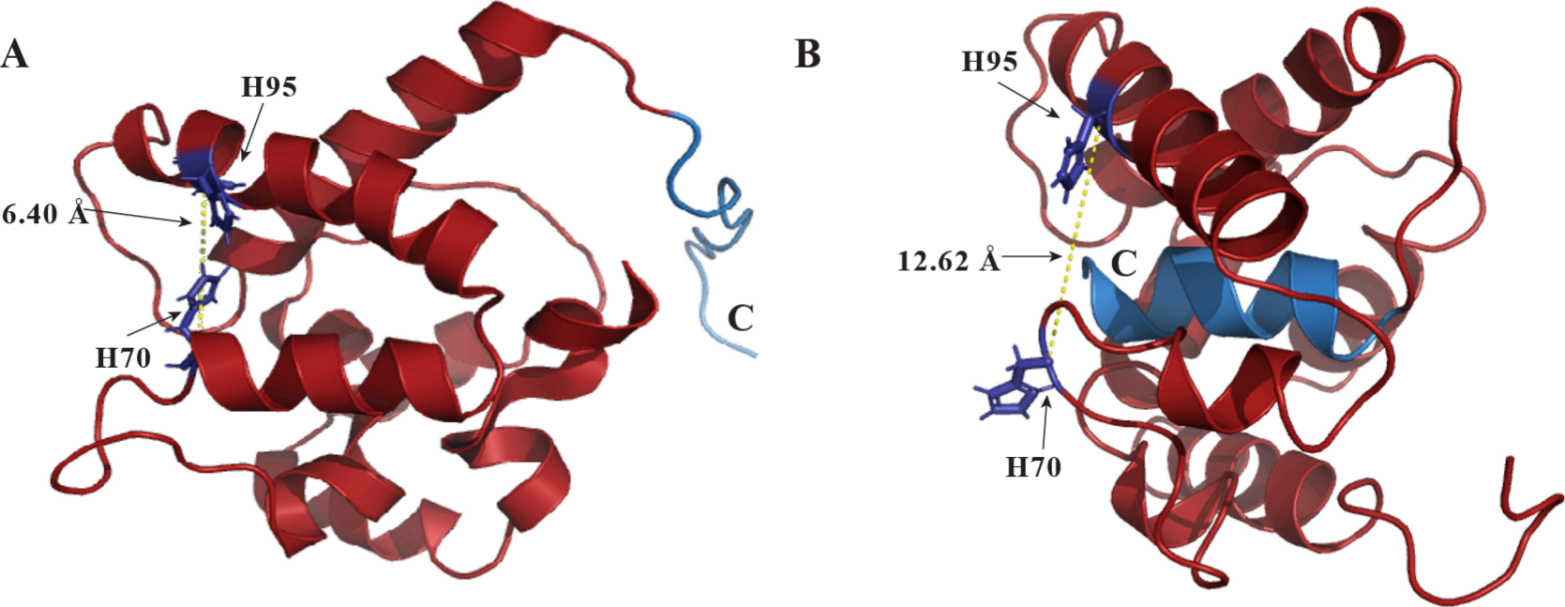
Representative comparison of histidine gates between open and closed conformations at different pH conditions. The protein backbone is in red, H residues in purple and the C-terminus in light blue. (A) NMR structure of ApolPBP1 (PDB ID: 1QWV) at pH 6.3, unstructured C-terminus and 6.40 Å between H70 and H95, (B) NMR structure of ApolPBP1 (PDB ID: 2JPO) at pH 4.5, C-terminal α-helix and 12.62 Å between H70 and H95. Adapted with permission under a Creative Commons CC-BY 4.0 (https://creativecommons.org/licenses/by/4.0/) from [128]. Colors and labels have been modified for clarity. Copyright 2009 ASBMB, currently Elsevier. Structural visualization was performed using PyMOL.^249^

**Figure 8:**
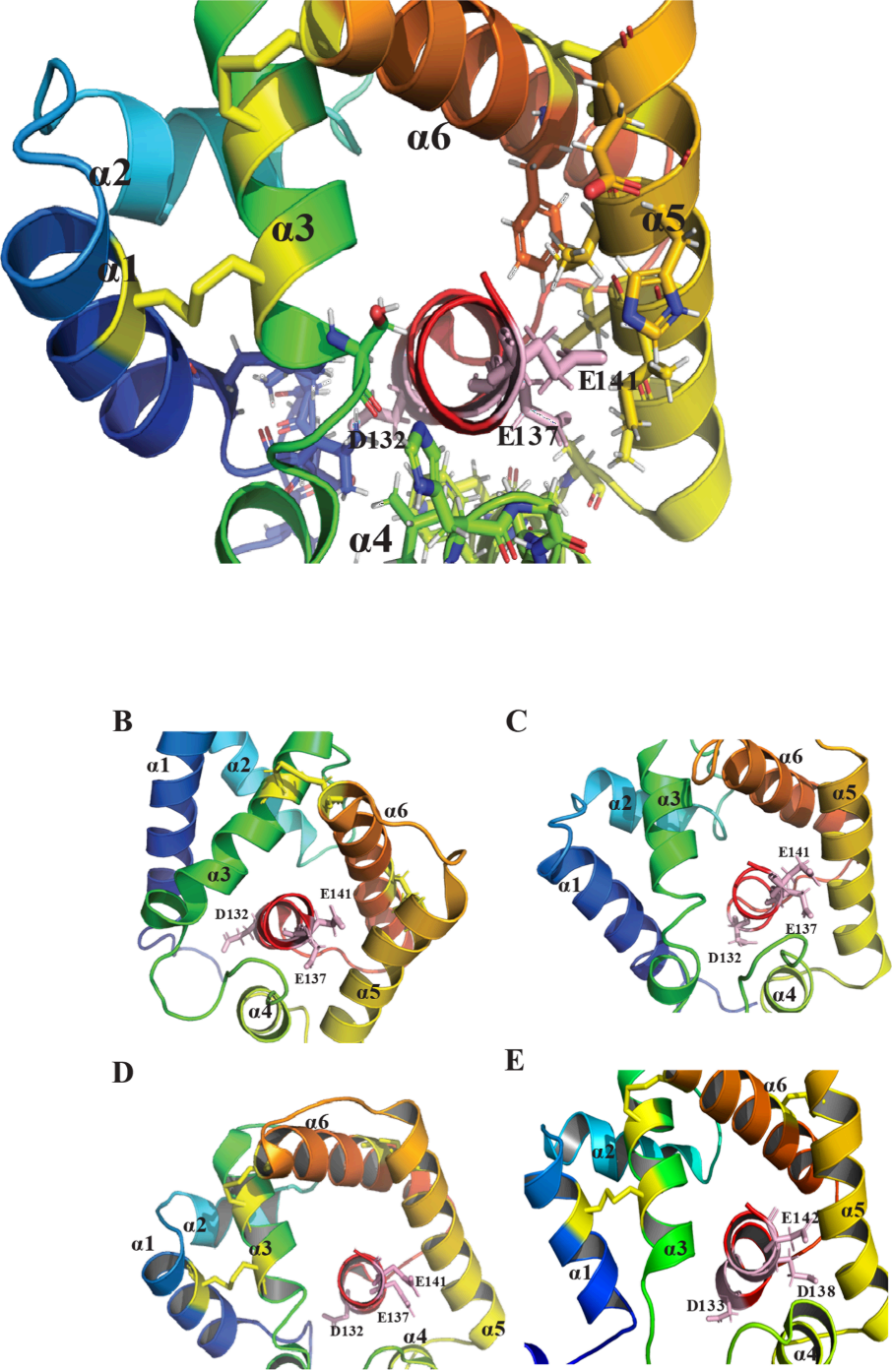
C-Terminal Helices of the A-Form PBPs with acidic residues in sticks: (A) BmorPBP1 (PDB ID: 1GM0) with interactions within 4 Å of acidic residues highlighted, (B) BmorPBP1 (PDB ID: 2FJY), (C) ApolPBP1 (PDB ID: 2JPO), (D) AtraPBP1 (PDB ID: 2KPH), (E) LdisPBP1 (PDB ID: 6UM9). Structural visualization was performed using PyMOL.^249^

C-terminal truncation experiments show variable effects on ligand binding across the PBPs. Truncation of the C-terminus in BmorPBP1 (ΔP129–V142) and AtraPBP1 enhances ligand binding at neutral pH, while similar truncations in ApolPBP1, CpomPBP2, and LdisPBP2 reduce binding affinity under the same conditions.^125,129,136^ Key acidic residues (Asp132, Glu137, Glu141) (Figure 8C) located in the C-terminus drive the reversible coil ⇔ helix transition, enabling conformational changes.^137^ For instance, a double mutation in ApolPBP1 acidic residues (E137Q/E141Q) abolishes this switch entirely, locking the protein in the open PBP^B^ state across all pH levels and impairing both ligand binding and release.^137^ While some residues are crucial for conformational dynamics, others appear dispensable. For example, tryptophan residues at positions 37 and 127 in BmorPBP1 do not affect binding to bombykol, indicating they may play more of a structural or stabilizing role and are likely targeted for degradation by odorant-degrading enzymes after ligand release.^138^ Beyond gating and structural transitions, key residues have been identified across PBPs that govern ligand-binding specificity^37,93,112,125,139^ (Figure 9 and Table 5). These findings deepen our understanding of the structural elements that regulate ligand binding and release while hinting at evolutionary adaptations in ligand recognition.

**Figure 9:**
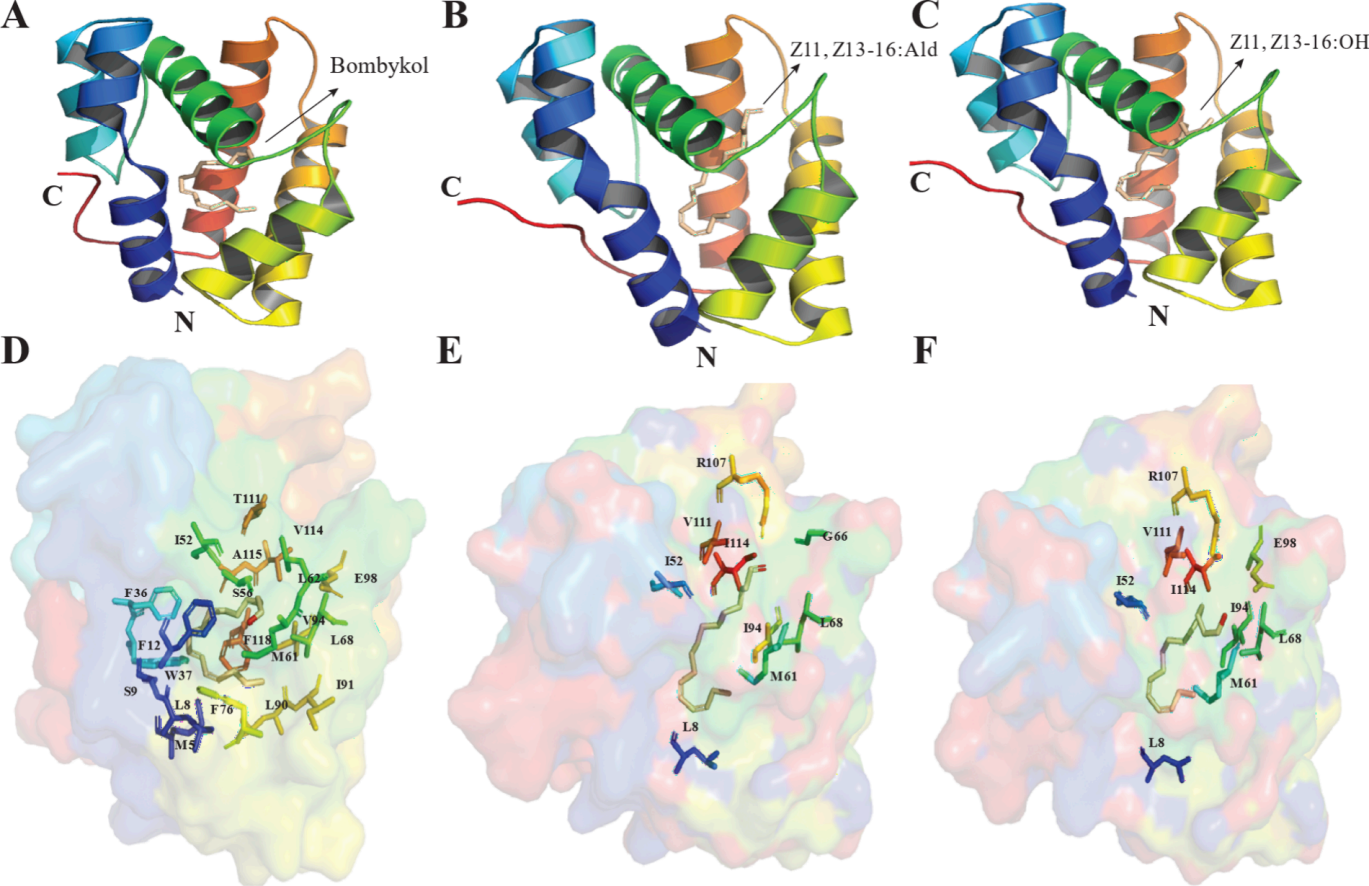
Ligand-Protein Interactions in Moth PBP-Ligand Complexes. The top figures present crystallographic structures of moth PBPs bound to their respective ligands with helices color-coded. The bottom figures highlight the key protein residues (stick models superimposed on surface depiction of protein) involved in ligand (stick models) binding. (A) BmorPBP1 complexed with bombykol (PDB ID: 1DQE, 1.80 Å resolution) at pH 8.0, (B) AtraPBP1 complexed with Z11, Z13–16: Ald (PDB ID: 4INW, 1.14 Å resolution) at pH 6.5, (C) AtraPBP1 complexed with Z11, Z13–16: OH (PDB ID: 4INX, 1.85 Å resolution) at pH 6.5, (D) Binding site residues of BmorPBP1 with bombykol, (E) Binding site residues of AtraPBP1 with the Z11,Z13–16:Ald, (F) Binding site residues of AtraPBP1 with Z11,Z13–16:OH. Structural visualization was performed using PyMOL.^249^

**Table 5:** Key Binding Residues and Interaction Types of Pheromone-binding Proteins (PBPs) With Their Respective Pheromones, Based On Experimentally Determined (PDB ID) or Homology-based Models

PBP	pheromone	PDB ID	key binding residues	interaction type
*Bombyx mori* PBP1 (BmorPBP/BmorPBP1)	*E*10, *Z*12–16: OH	1DQE	Ser56 (H-bond), Phe12, Phe118, Met5, Leu8, Ser9, Phe36, Trp37, Ile52, Met61, Leu62, Leu68, Phe76, Leu90, Ile91, Val94, Glu98, Thr111, Val114, Ala115 ^37^	hydrogen bonding (Ser56), hydrophobic interactions^37^
*Amyelois transitella* PBP1 (AtraPBP1)	*Z*11, *Z*13–16: Ald	4INW	Arg107 (H-bond), Met61, Gly66 (water-mediated), Leu8, Ile52, Met61, Leu68, Ile94, Val111, Ile114 ^93^	hydrogen bonding (Arg107), water-mediated interactions, hydrophobic interactions^93^
*Amyelois transitella* PBP1 (AtraPBP1)	*Z*11, *Z*13–16: OH	4INX	Met61 (H-bond), Arg107, Glu98 (water-mediated), Leu8, Ile52, Met61, Leu68, Ile94, Val111, Ile114 ^93^	hydrogen bonding (Met61), water-mediated interactions, hydrophobic interactions^93^
*Cydia pomonella* PBP1 (CpomPBP1)	*E*8, *E*10–12: OH	homology-based	Phe12, Trp37 ^125^	hydrophobic interactions^125^
*Athetis lepigone* PBP1 (AlepPBP1)	*Z*7–12: Ac	homology-based	Phe36, Trp37, Phe118 ^139^	hydrophobic interactions^139^
*Athetis lepigone* PBP1 (AlepPBP1)	*Z*9–14: Ac	homology-based	Phe36, Trp37, Val52, Phe118 ^139^	hydrophobic interactions^139^
*Tuta absoluta* PBP3 (TabsPBP3)	3*E*, 8*Z*, 11*Z:* Ac	homology-based	Phe37, Tyr61, Ile77, Leu84, Ile86, Leu87, Phe101, Ala136, Ile139, Ala140 ^112^	salt bridge (Arg135), hydrophobic interactions^112^

#### 2.3.3 Noncanonical Mechanisms

Interestingly, certain lepidopteran PBPs, such as *O. furnacalis* PBP2 (OfurPBP2) and *O. nubilalis* PBP3 (OnubPBP3), deviate from the canonical ligand release mechanism.^108,110^ For instance, they do not adopt a well-defined PBP^A^ conformation but assume a more fluid structural state, known as the molten globule (MG) state, as observed through NMR spectroscopy and far-UV CD studies.^108,110^ Notably, OfurPBP2 retains a structured C-terminal helix even at pH 6.5, unlike most PBPs^110^ (Figure 10A). Its gating mechanism involves an Arg70-His88 pair, functionally analogous to the His70-His95 gate in other PBPs^110^ (Figure 10B).

**Figure 10:**
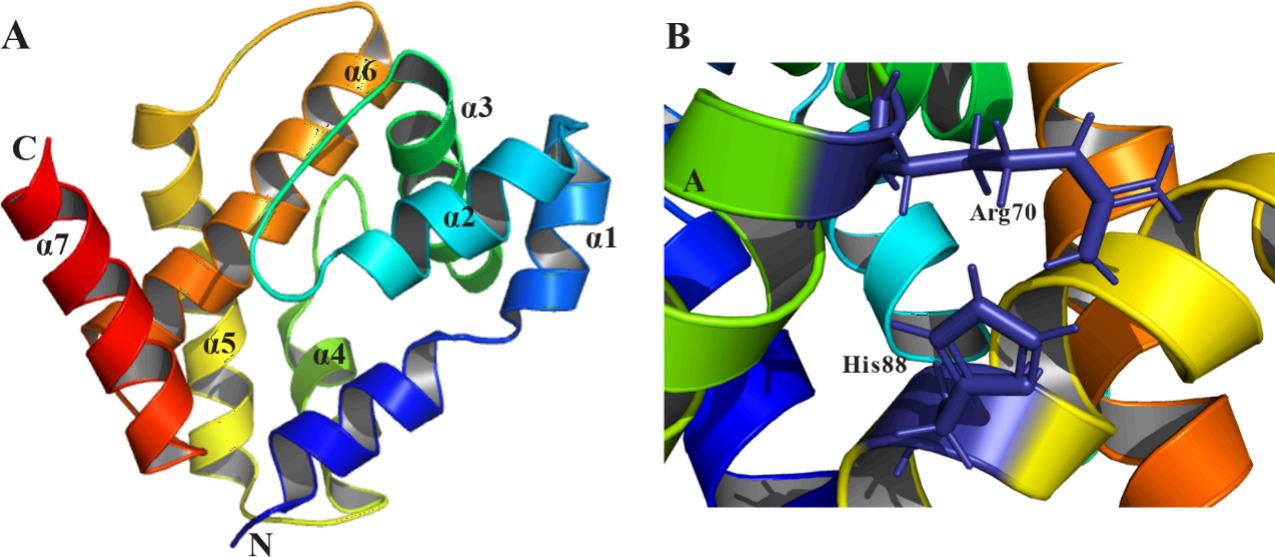
Averaged NMR structures of OfurPBP2 at pH 6.5 (PDB ID: 7UO6). Helices are colored from blue (N-terminus) to red (C-terminus): (A) with a well-structured C-terminal helix (α7), (B) Arg70-His88 gate shown as stick representations in violet. Structural visualization was performed using PyMOL.^249^

EposPBP3 exhibits disordered N- and C-termini, an intermediate state between two distinct conformations, forming a widely open internal cavity unlike the compact, closed cavities of other PBPs.^120^ Such structural adaptability suggests a potentially more promiscuous or dynamic binding mechanism, allowing EposPBP3 to modulate ligand affinity or specificity in response to environmental conditions.^120^ These noncanonical behaviors reveal evolutionary adaptations among PBPs and highlight new possibilities for synthetic ligand design aimed at pest control or biosensing.

### 2.4 Comparative Analysis across Other Insect Orders

While Lepidopteran PBPs have long served as a model system for understanding insect olfaction, PBPs are ubiquitous across insect taxa and display considerable variation in structure, ligand affinities, expression patterns, and functional roles. These differences reflect evolutionary adaptations to distinct ecological pressures and species-specific modes of chemical communication. For instance, lepidopteran PBPs typically exhibit high specificity for long-chain hydrophobic pheromones and undergo a pH-dependent conformational transition wherein a C-terminal α-helix folds into the ligand-binding pocket, facilitating ligand release near ORs.^37,108,128^ In contrast, Coleopteran PBPs, such as CbuqPBP2 in *Cyrtotrachelus buqueti*, retain the conserved six-cysteine framework and overall structural fold but exhibit a broader ligand repertoire that includes both pheromones and host plant volatiles.^140^ Furthermore, these PBPs bind ligands primarily through hydrophobic interactions with no evidence for pH-dependent ligand release.^140^ In Diptera, the *Drosophila* PBP LUSH not only binds the male-produced aggregation pheromone *cis*-vaccenyl acetate (cVA) but also acts as a molecular coactivator essential for receptor activation, underscoring a distinct functional role beyond mere transport.^10,141^ By contrast, PBPs in Blattodea, such as LmaPBP from *Leucophaea maderae*, are adapted to small, hydrophilic pheromones and possess a more open, water-accessible binding pocket, suggesting a release mechanism that diverges fundamentally from the pH-triggered model observed in moths.^142^ To elucidate these lineage-specific adaptations, we present in Table 6 a comparative overview of lepidopteran PBPs alongside those from three ecologically and phylogenetically diverse insect orders: Coleoptera (beetles), Diptera (flies), and Blattodea (cockroaches).

**Table 6:** Comparative Table of Pheromone-Binding Proteins across Insect Orders

feature	lepidoptera	coleoptera	diptera	blattodea
**canonical example**	BmorPBP1 (bombykol-binding)^132,37^	CbuqPBP2 (binds dibutyl phthalate, styrene)^140^	LUSH (binds cis-vaccenyl acetate, VA)^10,141^	LmaPBP (binds 3-hydroxy-butan-2-one)^142^
**ligand type**	long-chain hydrophobic compounds	volatile aromatic compounds and plant volatiles	short-chain alcohols/fatty acid derivatives (e.g., cis-vaccenyl acetate)	short-chain hydrophilic ketones and acids
**cysteine motif**	6 conserved cysteines: C1-X25–30-C2-X3-C3-X36–42-C4-X8-C5-X8-C6	same motif: C1-X15–39-C2-X3-C3-X21–44-C4-X7–12-C5-X8-C6	same general motif	same general motif (6 cysteines forming 3 disulfide bridges)
**structure (helices)**	6 α-helices, forms a hydrophobic binding pocket, C-terminal helix is dynamic and controls ligand access	6 α-helices, similar fold to Lepidoptera	6 α-helices, C-terminal is structured and forms part of the cavity; flexibility arises elsewhere	6 α-helices; lacks seventh helix, smaller and more hydrophilic cavity
**conformational change**	At low pH, C-terminal helix (seventh helix) folds into the binding cavity to release ligand	not reported	no seventh helix but C-terminal tail already folded into the core forming part of the binding site, conformational changes not pH-dependent; LUSH may act as receptor coactivator, not just transporter	no seventh helix; different mechanism than pH-triggered release
**expression bias**	typically, male-biased, expressed in trichoid sensilla	male-biased, expressed in antennae	expressed in both sexes; in trichoid sensilla	female-biased; key in female antennae for detecting male pheromones
**ligand binding mechanism**	hydrophobic interaction within deeply buried cavity	hydrophobic interactions	specific interaction with VA; acts as obligatory cofactor for neuronal activation	hydrophilic binding cavity: distinct mechanism adapted to polar ligands

## 3 Functional Differentiation and Ligand Specificity among PBP Paralogs in Lepidopteran Species

The presence of multiple PBP paralogs within conspecific species led to early hypotheses proposing functional differentiation among them, with each paralog exhibiting selectivity for distinct pheromone components or chemical properties.^48,143^ This idea is consistent with the complex, multicomponent nature of moth sex pheromones and supports the notion that PBPs contribute to signal specificity and fidelity during olfactory processing.^83^ However, emerging evidence also points to varying degrees of binding promiscuity among these paralogs, suggesting roles as general carriers or molecular buffers (i.e., proteins that modulate the availability of ligands) that amplify sensitivity or expand breadth of detection.^94,99,100,126,127,144–148^ Functional redundancy or partial overlap in ligand recognition could ensure robustness in pheromone detection, while hierarchical contribution where certain PBPs dominate the detection of primary components may refine olfactory processing.^94,99,100,126,127,144–148^

Traditionally, ligand-PBP interactions have been characterized using fluorescence-based competitive binding assays, employing fluorescent probes like *N*-phenyl-1-naphthylamine (1-NPN) or 1-aminoanthracene (AMA).^149,150^ These assays evaluate ligand binding affinity by measuring the displacement of fluorescent probes by candidate ligands (pheromones or analogs), allowing the calculation of dissociation constants (*K*_d_) that serve as quantitative indicators of binding strength.^129,138,149,150^ Reported affinities for PBP–ligand interactions typically fall within the micromolar (μM) range assuming the protein is fully active and follows a 1:1 binding stoichiometry.^99,119,126,129^ While variations in binding affinities among different ligands suggest a degree of ligand specificity, suggesting that PBPs may preferentially transport specific ligands to ORs, the binding affinities of ligands alone to PBPs do not consistently correlate with downstream physiological responses, such as electroantennogram (EAG) signals, or behavioral outcomes.^121,150,151^ This disconnect highlights the complexity of olfactory signaling, wherein factors such as receptor activation dynamics, ligand competition, and PBP diversity jointly shape the olfactory response.

Early studies supported the notion of high ligand specificity in PBPs, highlighting their ability to discriminate often distinguish among ligands based on structural features such as hydrocarbon chain length, stereochemistry, saturation, and functional groups (e.g., aldehydes, alcohols, esters).^108,117,136,138,139,152,153^ However, more recent evidence challenges this model of one-to-one specificity. Individual PBPs may exhibit varying degrees of selectivity, ranging from strong isomeric preferences to little or no selectivity, allowing them to bind and transport a variety of pheromone components.^122,126,149,154^ In some cases, PBPs exhibit selectivity for specific ligands, particularly in the presence of competing pheromone components^108,117,136,138,139,152,153^ (Table 7). In other instances, PBPs display more flexible and less specific binding profiles, accommodating a range of pheromone components^99,100,126,127,147,148^ (Table 8). However, increasing evidence points to a hierarchical organization among, where certain PBPs contribute more significantly to pheromone detection than others^94,127,144–146^ (Table 9). This is especially evident in species with multiple PBPs, where some proteins play primary roles in detecting key pheromone components, while others have more minor or redundant functions.^94,99,100,126,127,144–148^ This complexity suggests that pheromone detection is often mediated by an intricate network of interactions, in which multiple PBPs may share binding affinities for various pheromone components, contributing to a mixed pheromone blend perception.^83,85^

**Table 7:** Selectivity of PBPs for Specific Ligands

species	PBP	preferred ligands/binding specificity	notable findings
*Cydia pomonella*	CpomPBP1 ^136^	codlemone, 1-dodecanol, (*E, E*)-2,4-dodecadienal	shows high affinity for C12 compounds
*Bombyx mori* (Silkworm)	BmorPBP2 ^152^	bombykol	mediates response of BmOR1 to bombykol but not bombykal
*Antheraea pernyi*	AperPBP1,^149^ AperPBP3 ^149^	(*E, Z*)-6,11-hexadecadienyl acetate, (*E, Z*)-4,9-hexadecadienyl acetate	selective binding for specific acetates
*Antheraea pernyi*	AperPBP2 ^149^	(*E, Z*)-6,11-hexadecadienal	distinct preference for this aldehyde
*Ostrinia nubilalis*	OnubPBP3 ^108^	E-pheromone > Z-pheromone	greater stability with E-pheromone
*Lymantria dispar*	LdisPBPs^117^	specific enantiomers of pheromone components	preferential binding for specific enantiomers
*Cnaphalocrocis medinalis*	CmedPBP4 ^153^	*Z*11–16: Ald	unique binding pattern for this aldehyde
*Conogethes punctiferalis*	CpunPBP2 ^263^	*E*10–16:Ald, *Z*10–16:Ald >16:Ald	preference for both *E*10–16: Ald and *Z*10–16: Ald
*Grapholita funebrana*	GfunPBP2 ^264^	*Z*8–12: Ac, *Z*8–12: OH	strongest binding affinity among GfunPBPs
*Grapholita funebrana*	GfunPBP1.1 ^264^	*Z*8–14: Ac, *Z*10–14: Ac, 12: OH	higher binding affinity than other GfunPBPs for these components
*Grapholita funebrana*	GfunPBP1.2 ^264^	*E*8–12: Ac	exhibits stronger binding affinity to *E*8–12: Ac compared to others

**Table 8:** Flexible Binding Profiles of PBPs across Multiple Pheromone Components

species	PBP	preferred ligands/binding specificity	notable findings
*Bombyx mori*	BmorPBP/BmorPBP1 ^147^	nearly equal affinity for bombykol and bombykal	supported by *in vivo*,*in vitro* assays, and docking simulations
*Plutella xylostella*	PxylPBP3 ^99^	high affinity for both pheromones	pheromones may not share identical peri-receptor pathways
*Helicoverpa armigera*	HarmPBP1 ^148^	binds six pheromone components and analogs with equal affinity	no evident binding specificity
*Helicoverpa armigera*	HarmPBP2 ^148^	stronger binding to alcohols	lacks alcohol components in pheromone blend
*Helicoverpa armigera*	HarmPBP3 ^148^	stronger binding to acetates	lacks acetate components in pheromone blend
*Helicoverpa assulta*	HassPBP1 ^148^	binds six pheromone components and analogs with equal affinity	no evident binding specificity
*Helicoverpa assulta*	HassPBP2 ^148^	weaker affinity for acetates compared to HarmPBP2	uses *Z*11–16: Ald and *Z*9–16: Ald in its pheromone blend
*Helicoverpa assulta*	HassPBP3 ^148^	stronger binding to acetates	includes acetate components (*Z*11–16: Ac, *Z*9–16: Ac) absent in *H. armigera*
*Spodoptera litura*	SlitPBP1,^127,147^ SlitPBP2,^127,147^ SlitPBP3 ^127,147^	no distinct selectivity toward three primary pheromone components	supported by electrophysiological studies
*Chilo suppressalis*	CsupPBPs^100^	limited specificity	*in vitro* and *in vivo* studies confirm lack of strong binding discrimination
*Ostrinia furnacalis*	OfurPBPs^126^	no significant difference in binding affinity for *E*12–14: Ac and *Z*12–14: Ac	except OfurPBP4, which selectively binds *Z*12–14: Ac but not *E*12–14: Ac
*Ostrinia furnacalis*	OfurPBP2,^126^ OfurPBP4,^126^ OfurPBP5 ^126^	stronger affinities for *O. nubilalis* pheromones than its own	indicates potential cross-species interactions

**Table 9:** Hierarchical Contribution of PBPs to Pheromone Detection

species	PBP	preferred ligands/binding specificity	notable findings
*Spodoptera litura*	SlitPBP1 ^127,144,147^	stronger binding to sex pheromones	major contributor to pheromone perception, plays a more prominent role than SlitPBP2 and SlitPBP3
*Spodoptera litura*	SlitPBP2,^127,144,147^ SlitPBP3 ^127,144,147^	weaker binding	minor contributors
*Spodoptera inferens*	SinfPBP1 ^94^	high affinity for *Z*11–16: Ac, *Z*11–16: OH, *Z*11–16: Ald	key role in pheromone perception
*Spodoptera inferens*	SinfPBP3 ^94^	no notable binding	limited role
*Chilo suppressalis*	CsupPBP1,^145^ CsupPBP3 ^145^	high affinities for pheromone components	CsupPBP1 plays a more critical role, verified through *in vivo* functional studies
*Chilo suppressalis*	other CsupPBPs^145^	lower binding affinities	less significant in perception

The coexistence of multiple PBP paralogues with diverse, hierarchical, and sometimes overlapping ligand-binding profiles likely reflects species-specific evolutionary pressures and ecological demands. In some species, a broad binding profile may offer adaptive advantages by enabling the detection of a wider array of pheromone cues or environmental volatiles. In contrast, high ligand specificity may promote reproductive isolation or ensure precise communication. These findings highlight the variability and plasticity of pheromone recognition mechanisms across species, supporting a dynamic, integrative view of PBP function, shaped by molecular architecture, ecological pressures, and evolutionary history.

## 4 Lepidopteran PBP Kinetics

Much of the existing research on PBPs has focused on their ligand-binding affinities and structural characterization in apo- and ligand-bound conformations, whereas kinetic data remain sparse but conceptually important. Modeling data suggests that within approximately 3 ms of entering the sensillum lymph, about 17% of the total pheromone is enzymatically degraded, while the remaining 83% binds to PBPs, thereby largely shielded from enzymatic degradation.^6,155^ These figures were derived from detailed kinetic models, such as Kaissling’s Model N as well as others, which integrate perireceptor and receptor events, including pheromone uptake, diffusion, degradation, and PBP-mediated transport, to simulate the temporal dynamics of olfactory responses in moths.^6,155,156^ Although these models provide valuable insight into the complex extracellular environment of the olfactory sensillum, no studies to date have directly quantified the time required to reach equilibrium in PBP-ligand interactions, even though moths are known to respond to pheromone signals within milliseconds.^91,156^

The kinetics of PBP-ligand interactions remain relatively unexplored, though several studies have reported valuable data on the association and dissociation rate constants (*k*_on_ and *k*_off)_ for various PBP-pheromone pairs^91,138,157^ (Table 10). Notably, LdisPBP2 exhibits significantly slower association rates with both (+)-disparlure and (−)-disparlure enantiomers compared to other PBPs.^91^ These kinetic studies identified a rapid initial association event observed within the first 5 s, though available techniques at the time could not fully resolve this process.^91^ This was followed by a slower internalization of the ligand into the protein’s binding pocket, supporting a two-step ligand association mechanism.^91^ In this model, the pheromone first attaches to an external site on the PBP before being gradually accommodated into the protein’s binding pocket, suggesting that the initial fast step may facilitate the rapid detection of pheromones in the environment, even if final equilibrium binding is achieved over a longer time scale.^91^ It also raises the possibility that certain PBPs may have evolved intrinsically slower kinetics, not as a limitation but as an adaptive feature to buffer responses to persistent signals or to maintain prolonged stimulation from ecologically relevant ligands. Importantly, these kinetic features align with behavioral and computational evidence showing that insects can detect and respond to pheromones within subsecond timeframes. Thus, the primary rate-limiting step in olfactory signal transduction is unlikely to be initial pheromone binding, but rather downstream events such as ligand transfer to ORs and subsequent neuronal activation.^156,158^

**Table 10:** Kinetic Parameters of Pheromone-Binding Proteins (PBPs) across Different Species

PBP	pheromone	*k*_on_ (M^–1^s^–1^)	*k*_off_ (s^–1^)	notable insights
ApolPBP1 ^157^	*E*6, *Z*11–16: Ac	1.7 × 10^5^	0.01	high affinity ensures efficient pheromone uptake.
BmorPBP/BmorPBP1 ^138^	*E*10, *Z*12–16: OH	6.8 × 10^4^	0.007	lower rate constants than ApolPBP1, but pheromone uptake half-life was still consistent with the biological dynamics of pheromone association due to the high concentration of PBP in the lymph
Ldis PBP2 ^91^	(+)-disparlure	4.8 × 10^2^	4.7 × 10^–4^	slower association rate: supporting a two-step ligand internalization mechanism with an initial rapid interaction observed
Ldis PBP2 ^91^	(−)-disparlure	1.6 × 10^2^	5.0 × 10^–4^	slower association rate: supporting a two-step ligand internalization mechanism with an initial rapid interaction observed

## 5 *In Vivo* Roles of Lepidopteran PBPs in Olfactory Signaling and Behavior

PBPs constitute a versatile family of proteins with functional roles extending beyond their initial characterization as passive pheromone carriers.^35^ As molecular filters within the hydrophilic sensillar lymph, PBPs selectively bind specific pheromones while excluding structurally similar nontarget compounds, simultaneously shielding labile ligands from enzymatic degradation as well as premature volatilization to preserve their structural integrity during transit to ORs.^6,35^ The development of genome editing, such as zinc-finger nucleases (ZFNs), transcription activator-like effector nucleases (TALENs), and the CRISPR/Cas9 system, has enabled *in vivo* functional studies that elucidate the nuanced roles of PBPs in modulating behavior, temporal signal resolution, and mate-seeking efficiency across diverse lepidopteran species.^159,160^ For instance, CRISPR-Cas9-mediated knockout of BmPBP1 significantly reduced antennal sensitivity to bombykol and bombykal, although BmPBP1-knockout male antennae retained dose-dependent EAG responses markedly lower than those of wild-type males.^161^ This residual activity suggests the existence of alternative solubilization mechanisms, including potential nonspecific binding by other soluble proteins in the sensillar lymph or functional redundancy via coexpressed OBPs.^162^ Supporting this, studies in *A. polyphemus* indicate that certain olfactory tubules within sensilla trichodea directly contact OSN dendritic membranes, potentially enabling direct pheromone-receptor interaction in the absence of PBPs.^162,163^

Behaviorally, BmPBP1-knockout males exhibit delayed and impaired orientation toward pheromone sources, initiating source-seeking behavior less frequently.^161^ Although data on mating success remain limited, these results suggest a critical role for PBPs in reproductive behaviors. In another study, BmPBP1 knockout reduced the temporal sensory resolution of male antennae, impairing their ability to efficiently track pheromone plumes and resulting in longer times to locate both pheromone sources and female moths.^164^ BmPBP1 was also found to regulate the rapid termination of olfactory signals, a process essential for precise detection and behavioral coordination.^164^ These findings highlight PBP roles in olfactory resolution and response kinetics with potential applications in the development of artificial odor-tracking systems.

Additional studies reinforce the importance of the combinatorial PBP function. In a study in *H. armigera*, RNA interference (RNAi) silencing of both HarmPBP1 and HarmPBP2 reduced electrophysiological and behavioral sensitivity to the sex pheromone *Z*11-16: Ald, whereas silencing either gene individually had negligible effects.^165^ This suggests a combinatorial role for PBPs, supporting a redundant system that ensures robust pheromone detection across a range of environmental contexts. Further, diverse phenotypic outcomes have been observed in *S. litura*, *C. suppressalis*, *Spodoptera frugiperda*, *H. armigera*, and other species where CRISPR-Cas9-mediated knockout of multiple PBP genes resulted in markedly reduced antennal sensitivity and disrupted pheromone-mediated behaviors.^144–147,166–168^ While these findings reaffirm the critical role of PBPs in lepidopteran olfactory processing, they also provide insight into the specificity, selective affinity, and functional differentiation among PBP paralogs—revealing their contributions to odorant detection and pheromone discrimination within a species.^144–147,166–168^

In *S. frugiperda*, gene knockout of either SfruPBP1 or SfruPBP2 abolished male responses to key pheromone components (*Z*9-14: Ac and *Z*7-12: Ac), resulting in complete disruption of orientation, wing vibration, and courtship displays.^169^ These findings suggest a critical and possibly cooperative role for these PBPs in pheromone detection and behavioral activation. The authors postulate that SfruPBP1 and SfruPBP2 may form homo- or heterodimers, proposing a model in which PBP dimerization enhances the sensitivity and reliability of pheromone detection.^169^ Although first reported over a decade ago,^29^ scattered evidence supports the idea that PBPs in certain insect species can undergo dynamic oligomerization, forming dimers that modulate their ligand-binding and scavenging properties in more complex and context-specific ways than previously recognized.^37,91,170–172^ *In vitro* studies have shown that such dimerization can enhance functional capacity by modifying ligand-binding characteristics, potentially through conformational changes that generate novel hydrophobic tunnels or allosteric binding sites.^170–172^ Native PAGE experiments in LdisPBP1 and LdisPBP2 also reveal that both monomeric and aggregated (dimeric, multimeric) PBPs can bind pheromones, suggesting that at higher PBP concentrations, additional binding sites may become available at the interfaces of aggregated PBP monomers.^122^ This supports the view that several PBPs form active dimers and that such cooperative interactions may stabilize ligand binding, facilitate sequential handoff to ORs, or enhance ligand scavenging, ultimately contributing to improved signal fidelity and more efficient transduction.

Despite the advantages of gene-editing approaches such as CRISPR-Cas9, several challenges remain.^173^ Precision targeting is complicated by off-target effects, redundancy with other OBPs, and reduced viability of homozygous mutants.^174–177^ For example, off-target mutations may partially account for reduced survival rates and confound phenotypic analyses.^176,177^ Moreover, in highly redundant olfactory networks, single-gene knockouts often underestimate true functional contributions, necessitating multiple or combinatorial knockouts for definitive interpretation.^99,100,126,165^ Technical barriers, such as species-specific constraints on embryo manipulation and delivery of gene-editing components, also restrict the broader applicability of these tools.^173,174^

Beyond their canonical sensory roles, PBPs are also expressed in nonsensory tissues, where they likely aid in pheromone storage and controlled release. For instance, AaenPBP2 of *Agriphila aeneociliella* is highly expressed in both male antennae and abdomens than in females, implicating dual functions in pheromone detection and secretion.^178^ PBPs in secretory glands may also regulate the ratios of pheromone components through autocrine feedback loops to meet environmental or behavioral demands, offering a single-protein mechanism that circumvents complex enzymatic cascades.^179^ Collectively, these findings underscore the multifaceted roles of PBPs in pheromone detection, signal resolution, behavioral coordination, and possibly endocrine-like regulation. They also offer a blueprint for potential bioinspired applications, such as artificial odor-tracking systems and novel pest control strategies targeting olfactory communication pathways.

## 6 Interplay between PBPs, ORs, and SNMPs in Lepidopteran Olfaction

Beyond their role in transport, PBPs also likely participate in pheromone scavenging to prevent overstimulation of ORs and ensure activation is tightly regulated and transient.^6,13,91^ Kaissling’s model proposes that pheromone deactivation involves conversion of PBPs into a scavenger form, rendering the pheromone–PBP complex inactive and incapable of stimulating receptors.^6,155^ This transformation may be catalyzed by a hypothetical enzyme, by the receptor itself, or occur spontaneously.^80,138^ It likely accounts for the rapid termination of receptor potentials despite the slow degradation of pheromones.^6,155^

In addition, PBPs likely play a direct role in modulating the OR function. The first ORs identified in moths were BmorOR1 and BmorOR3 in *B. mori*, but other lepidopterans such as European corn borer, *O. nubilalis*, and the tobacco budworm, *H. virescens*, have been extensively researched for their pheromone detection and response mechanisms.^47,51,180–182^ Functional studies using heterologous expression systems like *Xenopus* oocytes or modified mammalian HEK (human embryonic kidney) cells have demonstrated that coexpression of PBPs with ORs significantly increases receptor sensitivity to pheromones compared to conditions where only hydrophobic solubilizers like dimethyl sulfoxide (DMSO) are used.^53^ This indicates that PBPs play a critical role in solubilizing hydrophobic pheromones into an aqueous environment, thereby enhancing receptor accessibility and sensitivity.^53^ Furthermore, PBPs modulate the selectivity of OR responses—an effect not replicated by DMSO—indicating that PBPs are not passive carriers but active modulators of signal specificity.^49,50,183^ For instance, in HEK293 cells expressing BmorOR1 or BmorOR3, BmorOR1 showed responses to both *E*10,*Z*12–16:OH and *E*10,*Z*12–16:Ald when delivered in DMSO.^50^ However, when recombinant BmorPBP1 was introduced with *E*10, *Z*12-16:OH, a significant activation of BmorOR1 occurred, while the same was not observed in combination with *E*10,*Z*12-16:Ald, indicating ligand-selective activation.^50^ Interestingly, the latter compound also failed to activate BmorOR3 (which is sensitive to it) in the presence of BmorPBP1, suggesting that PBPs confer receptor-specific ligand discrimination as well.^50^ Similar findings were observed in experiments with *H. virescens*, where HvirPBP1 and HvirPBP2 modulated responses of OR13 depending on the pheromone component tested.^49^ In *A. polyphemus*, distinct PBPs showed preferential binding to specific pheromone components, and their presence altered receptor responses, suggesting that PBPs play a role in fine-tuning pheromone detection.^183^

In *P. xylostella*, PBPs not only enhanced receptor sensitivity but also influenced which components were recognized by the receptors, leading to variations in activation strength.^99,184^ This effect was also observed in a study of *C. suppressalis* ORs expressed in *Xenopus* oocytes, as well as in the ORs of related species *H. armigera* and *H. assulta*. In these cases, PBPs modulated receptor activity in response to specific pheromone blends and analogs, further supporting the idea that PBPs help fine-tune olfactory signaling.^53,100^

Adding to this complexity is the role of SNMPs, particularly SNMP1, that form a tripartite complex with PBPs and ORs, facilitating the rapid activation and deactivation of ORs by enabling efficient ligand delivery and release.^67,185^ Mechanistically, SNMP1 is thought to act as a docking or coreceptor protein with its ectodomain serving as a tunnel-like structure, shuttling ligands into the lipid membrane or the receptor pocket, shielding them from degradation and possibly triggering their release from PBPs via local pH changes or protein–protein interactions, functions analogous to scavenger receptors like CD36.^67,185^

Reconstitution experiments confirm that SNMP1 enhances OR responsiveness in heterologous systems.^67^ In HEK293 cells, coexpression of HvirSNMP1 with the HR13 receptor increased sensitivity to (*Z*)-11-hexadecenal by ∼1000-fold.^186^ Similarly, *H. armigera* SNMP1 knockouts exhibited reduced EAG responses to pheromones and diminished mating success, while responding normally to plant volatiles.^187^ Notably, HarmSNMP1–/–mutants exhibited impaired detection of long-chain but not short-chain pheromones, suggesting ligand-length specificity.^187^ Yeast two-hybrid assays demonstrated physical interactions between SNMP1 and both ORs (e.g., HarmOR13, BmorOR1) and the coreceptor Orco in species such as *B. mori* and *H. armigera*.^188,189^ RNAi-mediated silencing of *B. mori* SNMP1 prolonged the time males took to locate females, emphasizing its behavioral relevance.^188^ Beyond delivery, SNMP1 likely aids in ligand clearance to reset the receptor complex, a critical function for insects navigating transient pheromone plumes.^67^ SNMP2 may assist by removing pheromone degradation products like fatty acids, helping maintain high temporal resolution.^67^ This model is supported by accelerated OR complex response kinetics (activation and deactivation) in *Xenopus* oocyte systems expressing BmorSNMP1.^190^

Altogether, pheromone detection in insects involves a finely orchestrated collaboration among PBPs, the OR/Orco complex, and SNMPs. PBPs are no longer viewed merely as solubilizers or transporters, but as active gatekeepers that modulate both receptor activation and ligand specificity.^76,164^ The involvement of SNMPs in the olfactory signaling model highlights a coordinated molecular triad and expands the framework into a sophisticated molecular relay system, wherein PBPs deliver odorants, SNMPs facilitate receptor activation and signal reset, and ORs detect the ligand, all within milliseconds, to ensure accurate, rapid, and dynamic pheromone signaling.^5,185^

## 7 Applications of PBPs in Pest Management and Biodiversity Conservation

Recent advances in molecular biology and structural biochemistry have enabled the development of synthetic pheromone mimetics that specifically bind to PBPs.^77,191^ These reverse chemical ecology strategies harness PBP binding affinities to screen and identify behaviorally active or disruptive ligands, offering sustainable, species-specific pest control solutions that align well with integrated pest management (IPM) principles.^15,191–193^

For example, structure-based screening through*in silico* molecular docking identified codlemone as a potent synergist for *Grapholita molesta*, increasing trap efficacy by up to 6-fold.^194^ This success reflects broader methodological advances across insect systems and is supported by structural studies of PBPs, such as those in *Bombyx mori*, highlighting the versatility of PBPs as platforms for semiochemical discovery.^98,195^ While *Bactrocera dorsalis* is not a lepidopteran, it provides a valuable methodological precedent, demonstrating an integrated workflow that combines computational docking, fluorescence-quenching assays, and behavioral validation, an approach that is readily transferable to lepidopteran PBPs.^192,196,197^ By disrupting mating, host-seeking, and oviposition behaviors, PBP-targeted strategies can augment pheromone-based interventions, including traps, bait systems, and mating disruption, thereby enhancing the effectiveness of pest management tools.^15,77,198^

PBP-targeted mimetics do not necessarily replicate behavioral signals but instead act by saturating or disrupting the ligand–PBP interaction, thereby impairing proper ligand delivery and potentially misactivating downstream olfactory pathways. For example, fluorescence displacement binding assays in *P. xylostella* (diamondback moth) reveal that PxylPBPs exhibit strong affinity for synthetic pheromone analogs containing double bonds, indicating competitive interference with natural pheromones.^99^ However, EAG recordings show limited male antennal responses to these analogs compared to natural pheromones, suggesting that synthetic compounds can saturate PBPs without eliciting mating behaviors, a strategic advantage in disrupting reproduction.^99^ Similar antagonistic effects have been observed across the species. In *O. nubilalis*, adding 1% (*Z*)-11-hexadecenal from *Sesamia nonagrioides* to its pheromone blends reduces male captures by 90% and pheromone contact by 83%, indicating potent antagonistic effects.^199^ Similarly, in *Cryptoblabes gnidiella*, stable analogs such as (*Z*)-9-tetradecenyl formate and (*Z*)-11-hexadecenyl formate act as potent behavioral disruptors, significantly reducing male attraction to both synthetic pheromones and calling females in wind tunnel tests, as well as decreasing male captures in pheromone-baited field trials.^200^ Such “decoy ligands” represent a promising strategy for selectively disorienting pest species while minimizing impacts on nontarget and beneficial insects.

In *L. dispar*, both short- and long-term electrophysiological inhibition of pheromone responses have been observed with synthetic compounds that reduce antennal sensitivity, including commercial repellents like DEET and novel benzene derivatives.^201^ In *Cydia pomonella*, novel nonfluorinated electrophilic keto derivatives that are structural analogs of native pheromones have been shown to antagonize pheromone responses by inducing erratic male flight in wind tunnel tests and reducing male captures in the field.^202^ Similarly, in *Ectropis grisescens*, a novel pheromone analog (*Z*3, *Z*6, *Z*9-19: Hy) significantly suppresses male behavioral responses in both EAG and wind tunnel experiments. This targeted disruptive effect is further supported by molecular docking studies.^203^ Likewise, in *Zeuzera pyrina*, various trifluoromethyl ketones (TFMKs) analogs significantly inhibit pheromone responses as demonstrated across EAG, wind tunnel, and field assays.^204^

These synthetic mimetics and behavioral antagonists can be deployed through various strategies, such as mating disruption, in which the environmental is saturated with synthetic mimetics to interfere with natural mating, feeding, and oviposition behavior.^198,205^ Additional approaches include attract-and-kill systems, which lure pests into traps containing toxic agents for targeted elimination.^205,206^ Genetic engineering tools such as CRISPR/Cas9 offer advanced opportunities to manipulate insect olfactory pathways, rendering pests less responsive to natural pheromones or more sensitive to synthetic cues, as demonstrated in *S. frugiperda*.^191,198,207^ In parallel, gene-silencing technologies like Spray-Induced and Nanoparticle-Delivered Gene Silencing (SI-NDGS) provide nontransgenic alternatives, involving the exogenous application of double-stranded RNA (dsRNA) to target essential pest genes, including those involved in pheromone detection.^198,208^ For instance, RNAi targeting *EcR* in *S. frugiperda* and several Hemipteran rice pests (*Nilaparvata lugens*, *Sogatella furcifera*, *Laodelphax striatellus*) impairs oviposition, host-seeking, and mating behaviors, ultimately reducing reproductive success.^209–212^ Nanoparticle-based delivery systems further enhance dsRNA stability, cellular uptake, and environmental persistence, enabling a precise, nontoxic, and species-specific pest control. These merging tools pave the way for developing pest-resistant crops that incorporate pheromone-based disruption at the molecular level.^208,209^

Beyond their role in pheromone interference, PBPs are being increasingly repurposed for biosensor development in pest detection and surveillance.^191,213^ PBP-based biosensors, such as field-deployable electronic noses and lab-on-chip systems, leverage the high affinity and specificity of PBPs for volatile semiochemicals to enable real-time monitoring of pest presence at early infestation stages.^213,214^ These technologies can provide timely data for IPM decisions. For example, recombinant ORs/PBPs immobilized on sensor chips have been used successfully to detect specific pheromone components in *H. armigera*, *Ectropis obliqua*, and *B. mori*, demonstrating the feasibility of real-time biosensing applications in agricultural settings.^215–218^

These applications also hold promise for biodiversity conservation. For example, the use of species-specific pheromone mimetics to target invasive pests like *S. frugiperda* can minimize the collateral effects on beneficial insects and help preserve native ecosystems.^219^ However, translating laboratory findings into effective field applications remains challenging. Natural environments introduce numerous variables, such as fluctuating temperatures, humidity, wind patterns, and background volatiles, that influence pheromone dispersion, volatility, and PBP-pheromone interactions.^220,221^ Additionally, scalability presents a significant barrier as the synthesis of high-purity, enantiomerically specific pheromone analogs with strong binding affinity to PBPs is often cost-prohibitive at commercial levels.^222,223^ The logistical demands of large-scale deployment also pose significant challenges, requiring substantial material input and coordinated release mechanisms (e.g., aerosol devices, dispensers, or pheromone-infused biodegradable matrices) to maintain consistent pheromone plumes across diverse and variable landscapes.^15,222,223^

These financial and operational constraints restrict the widespread use of pheromone-based strategies in high-value crops or tightly managed agricultural systems. However, advances in biotechnological production, automated release mechanisms, supply chain optimization, and regulatory harmonization offer promising avenues to broaden access. Even so, adoption is likely to remain uneven, with initial implementation concentrated among specialty producers. Integrating pheromone-based tools into comprehensive Integrated Pest Management (IPM) programs supported by enabling policies, farmer training, and investment in infrastructure will be critical to expanding their effective and economical use across diverse agricultural landscapes.

Despite their efficacy, the widespread adoption of pheromone-based tools remains constrained by high initial costs, technical complexity, and logistical challenges, limiting their practical use primarily to high-value or highly coordinated cropping systems. However, advances in biotechnological production, automated release technologies, supply chain optimization, and regulatory harmonization offer promising avenues for a broader deployment. Nonetheless, adoption is likely to remain uneven, with greater uptake among large-scale and specialty crop producers. Integrating pheromone-based strategies into comprehensive integrated pest management (IPM) frameworks—supported by enabling policies, capacity-building initiatives, and infrastructure investment—will be essential to enhance their accessibility, scalability, and cost-effectiveness across diverse agricultural systems.

Moreover, many pheromone analogs are prone to rapid degradation under ultraviolet (UV) radiation or microbial activity, limiting their field longevity.^220^ Advanced encapsulation technologies, including nanocarriers and slow-release polymers, can improve compound stability, extend lifespan, and prolong field efficacy, though these approaches often increase both cost and operational complexity as well as ecological considerations.^208^ The bioactivity of pheromone mimetics often depends on subtle structural fidelity, and field trials frequently produce variable outcomes. For example, while mating disruption has been shown to reduce male trap captures in *C. pomonella* and *P. xylostella*, corresponding decreases in crop damage and pest population levels have been inconsistent.^223–226^ Moreover, pests with high fecundity or strong migratory behavior, such as *S. frugiperda*, can rapidly recolonize treated areas, undermining long-term control measures.^227^ These limitations underscore the importance of developing region-specific strategies and integrating PBP-based interventions within broader multifaceted IPM frameworks.

Ecological considerations also warrant attention as the specificity of PBPs is not absolute. Insect control strategies targeting chemoreception—including those involving synthetic pheromones—may carry unintended consequences that remain largely understudied.^228,229^ Structural similarities among pheromone components across taxonomically related species may affect partial cross reactivity with nontarget insects, including pollinators, parasitoids, and natural predators.^228,229^ For example, surveys conducted in Hungary and Ukraine found that traps baited with pest-specific pheromones also attracted nontarget noctuid moths.^230^ Similarly, a study on conservation-relevant burnet moths demonstrated that even monitoring lures can alter male mating behavior.^231^ Formulation strategies such as microencapsulation, though intended to enhance pheromone stability and prolong release duration, may inadvertently exacerbate nontarget effects. By closely mimicking natural cues in both the chemical composition and release dynamics, these formulations can increase the likelihood of attracting unintended species. In 2012, approximately 2000 honeybee hives in Uruguay experienced mass mortality after foragers collected microencapsulated parathion-methyl, mistaking it for pollen.^232^ The pesticides had been applied 1–2 km away, demonstrating how visually and chemically deceptive formulations can pose severe risks to nontarget species. Once brought into the hive, the toxic capsules caused acute poisoning and colony collapse.^232^ This incident underscores the need for careful assessment of synthetic pheromone formulations and application practices to protect pollinators.

In addition to targeting mating behaviors, synthetic pheromone saturation in the environment may disrupt other critical olfactory-mediated functions such as host-seeking, foraging, and orientation—particularly in insects that depend on precise chemical cues for survival. Pollinators and parasitoids, for example, navigate complex odor landscapes to locate floral resources or hosts, and pervasive pheromone signals may induce olfactory confusion, reduce foraging efficiency, impair reproductive success, or diminish overall fitness. Such disruptions can cascade through ecological networks, altering predator–prey dynamics and compromising ecosystem resilience.

Notably, analogous effects have been documented outside insect systems; in invasive sea lampreys, synthetic pheromone cues were shown to displace native species’ reproductive behaviors by eliciting misleading female responses.^233^ Similarly, background pesticide odors and other sources of olfactory pollution have been found to impair bumblebee foraging accuracy and interfere with mating behaviors in moths, highlighting a broader vulnerability of odor-guided navigation systems to chemical interference, including from pheromone-based tools.^234,235^ Even plant-derived biopesticides, often considered more environmentally benign, have been shown to exert both lethal and sublethal effects on bees, predators, and parasitoids via their volatile constituents.^236,237^

Despite these emerging concerns, systematic evaluations of the ecological consequences of pheromone-based pest control remain scarce. Few studies have rigorously assessed the long-term impacts of synthetic pheromones on insect biodiversity, interspecific interactions, or essential ecosystem services, such as pollination and biological control. This significant knowledge gap underscores the need for comprehensive environmental risk assessments that incorporate nontarget organism behavior, community-level dynamics, and formulation-specific exposure risks.

Moreover, prolonged exposure to synthetic pheromones and pheromone-contaminated environments can contribute to human-induced rapid environmental change (HIREC), imposing evolutionary pressure on pest populations. This may lead to behavioral resistance or molecular adaptations, including shifts in mating rhythms or the adoption of alternative strategies involving PBPs.^238–240^ A recent study, the first to investigate alternative mating behaviors in insects under prolonged mating disruption, reported that male pink bollworm moths from exposed populations were significantly more likely to disrupt mating pairs than naïve males.^241^ This behavior appears to be an adaptive strategy to locate and access receptive females in environments saturated with synthetic pheromones.^241^ Similarly, environmental and ecotoxicological studies of large-scale ds-RNA applications to silence pest genes remain limited due to concerns regarding their safety and exposure risks to nontarget organisms like soil microbiota and aquatic fauna.^242^

Therefore, rigorous ecological risk assessments are essential to guide the responsible application of PBP-targeted technologies. These should include cross-species binding assays, sublethal behavioral evaluations, and assessments of multitrophic ecosystem impacts. Establishing safety thresholds and long-term environmental monitoring will be critical to ensure minimal disruption to nontarget organisms. Ultimately, the sustainable and effective deployment of these molecular innovations for pest management will depend on their integration into region-specific, ecologically grounded IPM frameworks.

## 8 Future Directions and Opportunities

Ongoing research into the genetic and molecular mechanisms underlying PBPs' functions will provide valuable insights into their evolutionary origins and functional diversity, as well as enhance the precision and sustainability of pheromone-based pest management. Advanced gene-editing and silencing techniques, including TALEN, RNAi, and CRISPR-Cas9 gene editing, enable precise manipulation of PBP expression, allowing researchers to assess phenotypic, electrophysiological, and behavioral changes in mutants exposed to specific semiochemicals. Phylogenetically informed experimental designs can further reveal adaptive signatures associated with host shifts, mating systems, or environmental variability. In addition, targeted gene knockouts and site-directed mutagenesis offer valuable tools for dissecting the molecular determinants of pheromone recognition, providing deeper insight into species specificity, evolutionary divergence, and potential resistance mechanisms. Complementary field studies examining the relationship among PBP expression levels, environmental variables, and ecological interactions will help elucidate the adaptive significance of these proteins. By integrating behavioral assays with molecular and genetic data, researchers can establish direct functional links between PBP function and moth behavior, enhancing our understanding of the critical role of PBP in chemical communication and ecological adaptation.

On the technological front, advances in structural modeling and molecular dynamics simulations are poised to resolve long-standing questions about the transient conformational states of PBPs during ligand entry and release.^111,243–246^ By systematically modulating structural features of candidate pheromone mimetics, including carbon chain length, degree of saturation, functional groups, hydrogen-bonding potential, and aromaticity, a rational design framework can be established. Furthermore, computational docking combined with molecular dynamics simulations enables a detailed investigation of how these modifications affect binding affinity and receptor activation, thereby guiding the development of highly stereospecific and selective analogs.^246^

Recent studies on quinoxaline, pyrimidine, and thiazolidinone scaffolds in cowpea aphids demonstrate that rational substitutions, particularly the incorporation of electron-withdrawing groups like chlorophenyl or sulfonates, may enhance binding affinity and insecticidal activity.^247^ In parallel, structure-based virtual screening (SBVS) efforts targeting *S. littoralis* olfactory receptors demonstrate that the feasibility of computational screening of natural product libraries can identify novel ligands, even in unexplored chemical space.^246^ This approach led to the discovery of two new ligands, which were subsequently validated using in vivo electrophysiology and behavioral assays.^246^ Furthermore, refinement of scoring functions, such as normalizing ligand efficiency (LE) to mitigate size bias, significantly improved the accuracy of agonist prediction, providing valuable insights for the optimization of future docking workflows.^246^

Isomerism and stereospecificity play critical roles in pheromone signaling and represent powerful tools for improving species-specific targeting in pest management. There is substantial interspecific variation in olfactory systems not only among lepidopteran species (e.g., *Pectinophora gossypiella* vs *B. mori*) but also across other orders, such as Hymenoptera (e.g., honeybees), to allow for the development of analogs that are selectively disruptive. This specificity is key to minimizing off-target effects and preserving ecologically and economically important pollinators. This was demonstrated in *Anopheles gambiae*, where virtual screening of essential oil compounds revealed selective inhibitors of OBP1 with stable interactions in the binding pocket and high binding free energies; such natural-product-inspired approaches could inform the design of pheromone mimetics.^248^ Future studies should explore adaptive responses to prolonged pheromone exposure through integrative approaches that combine behavioral assays with transcriptomic and proteomic profiling. These studies may uncover plasticity- or selection-driven changes in pheromone detection pathways, helping to preempt or mitigate the development of behavioral resistance. In parallel, long-term ecological assessments are essential to understand how large-scale manipulation of PBPs influences trophic interactions, pollinator populations, and overall biodiversity. Together, these research directions can guide the next generation of environmentally conscious, species-specific pest control strategies grounded in ecology. Looking ahead, bioinspired technologies, from odor-tracking drones to artificial noses, stand to benefit from the integration of PBP-based components capable of ultrafast and highly specific volatile detection in complex environments. Fusions of PBPs with optogenetic or electrochemical modules could create hybrid biosensors that match or even exceed insect olfactory sensitivity, with potential applications in environmental monitoring, food safety, and medical diagnostics.

In conclusion, PBPs play a central role in insect olfactory communication, mediating critical behaviors such as mate recognition, host location, and oviposition. Structural and functional studies have revealed their remarkable specificity, binding efficiency, and protective functions, highlighting their importance in pheromone transport and signaling. Beyond their endogenous biological roles, PBPs offer promising avenues for sustainable pest management and biodiversity conservation by enabling species-specific, ecologically responsible alternatives to conventional chemical controls. Continued research into the molecular dynamics, ecological roles, and translational potential of PBPs will not only deepen our understanding of olfactory signaling but also catalyze innovations in species-specific pest control and biosensor development. Bridging fundamental science and applied technology could have far-reaching impacts on sustainable agriculture, ecological management, and bioinspired engineering.

## Abbreviations

PBP: pheromone-binding protein
OBP: odorant-binding protein
GOBP: general odorant-binding protein
ABP: antennal binding protein
TALEN: transcription activator-like effector nuclease
CRISPR/Cas9: clustered regularly interspaced short palindromic repeats/CRISPR-associated protein 9
OSN: olfactory sensory neuron
ORN: olfactory receptor neuron
PDE: pheromone-degrading enzyme
OR: olfactory receptor
Orco: olfactory co-receptor
SNMP: sensory neuron membrane protein
GPCR: G-protein-coupled receptor
PLC: phospholipase C
PIP2: phosphatidylinositol 4,5-bisphosphate
IP3: inositol 1,4,5-trisphosphate
1-NPN: *N*-phenyl-1-naphthylamine
DAG: diacylglycerol
cAMP: cyclic adenosine monophosphate.
cGMP: cyclic guanosine monophosphate
HSQC NMR: heteronuclear single quantum coherence nuclear magnetic resonance
AMA: 1-aminoanthracene
CD: circular dichroism
EAG: electroantennogram
HEK: human embryonic kidney
DMSO: dimethyl sulfoxide
RNAi: RNA interference
SI-NDGS: spray-induced and nanoparticle-delivered gene silencing
TALEN: transcription activator-like effector nuclease
TFMK: trifluoromethyl ketone (pheromone analog)
ZFN: zinc-finger nuclease
GR: gustatory receptor
IPM: integrated pest management
CD36: cluster of differentiation 36
cAMP: cyclic adenosine monophosphate
EcR: ecdysone receptor
dsRNA: double-stranded RNA
*A. aeneociliella*: *Agriphila aeneociliella*
*A. pernyi*: *Antheraea pernyi*
*A. polyphemus*: *Antheraea polyphemus*
*A. transitella*: *Amyelois transitella*
*B. mori*: *Bombyx mori*
*C. suppressalis*: *Chilo suppressalis*
*C. pomonella*: *Cydia pomonella*
*C. punctiferalis*: *Conogethes punctiferalis*
*C. medinalis*: *Cnaphalocrocis medinalis*
*D. melanogaster*: *Drosophila melanogaster*
*E. postvittana*: *Epiphyas postvittana*
*H. armigera*: *Helicoverpa armigera*
*H. assulta*: *Helicoverpa assulta*
*H. cunea*: *Hyphantria cunea*
*H. virescens*: *Heliothis virescens*
*H. zea*: *Helicoverpa zea*
*L. dispar*: *Lymantria dispar*
*L. monachal*: *Lymantria monachal*
*M. sexta*: *Manduca sexta*
*O. furnacalis*: *Ostrinia furnacalis*
*O. nubilalis*: *Ostrinia nubilalis*
*P. xylostella*: *Plutella xylostella*
*S. frugiperda*: *Spodoptera frugiperda*
*S. inferens*: *Sesamia inferens*
*S. litura*: *Spodoptera litura*
